# Cerebral endothelial cell-derived extracellular vesicles regulate microglial polarization and promote autophagy via delivery of miR-672-5p

**DOI:** 10.1038/s41419-023-06173-5

**Published:** 2023-09-29

**Authors:** Changshui Wang, Lei Feng, Li Zhu, Linlin Wu, Beibei Chen, Changmeng Cui, Mengqi Yang, Yahao Gao, Pei Jiang

**Affiliations:** 1 Department of Neurosurgery, Affiliated Hospital of Jining Medical University, Jining Medical University, Jining, 272000 China; 2 Department of Neurosurgery, Jining First People’s Hospital, Shandong First Medical University, Jining, 272000 China; 3 Translational Pharmaceutical Laboratory, Jining First People’s Hospital, Shandong First Medical University, Jining, 272000 China; 4 Department of Oncology, Tengzhou Central People’s Hospital, Jining Medical University, Zaozhuang, 277500 China; 5 ADFA School of Science, University of New South Wales, Canberra, ACT Australia; 6 Institute of Translational Pharmacy, Jining Medical Research Academy, Jining, 272000 China

**Keywords:** Autophagy

## Abstract

The interaction between cerebral endothelial cells (CEC) and brain parenchymal cells is critical to maintain neurovascular homeostasis, whereas extracellular vesicles (EVs) are essential to mediate the cell–cell communication. Previous researches demonstrated that CEC-derived EVs (CEC-EVs) confer neuroprotective actions. However, the molecular mechanisms remain unknown. In this study, we isolated EVs from CEC and assessed their immune-regulatory actions in microglial cells and mice following lipopolysaccharide (LPS) exposure. We found that CEC-EVs treatment significantly ameliorated LPS-induced inflammatory activation, shifting microglial polarization from pro-inflammatory phenotype to anti-inflammatory phenotype. Meanwhile, microglial cells can effectively internalize CEC-EVs and this process was further enhanced by immune activation. Next, the miRNA microarray analysis revealed that CEC-EVs increased expression of miR-672-5p, which was demonstrated to be the cargo of CEC-EVs. TGFβ-activated kinase 1 (TAK1)-binding proteins 2 (TAB2) was identified to be the target of miR-672-5p. Through inhibiting TAB2, miR-672-5p derived from CEC-EVs suppressed TAK1-TAB signaling and thereby mitigating the downstream NF-κB activation. Furthermore, we found that by delivering miR-672-5p, CEC-EVs promoted autophagy and hence stimulating autophagic degradation of NLRP3 inflammasome. Our work firstly revealed the neuroimmune-modulating actions of CEC-EVs and further demonstrated that miR-672-5p secreted from CEC-EVs inhibits microglial pro-inflammatory polarization and facilitates autophagic process via targeting TAB2.

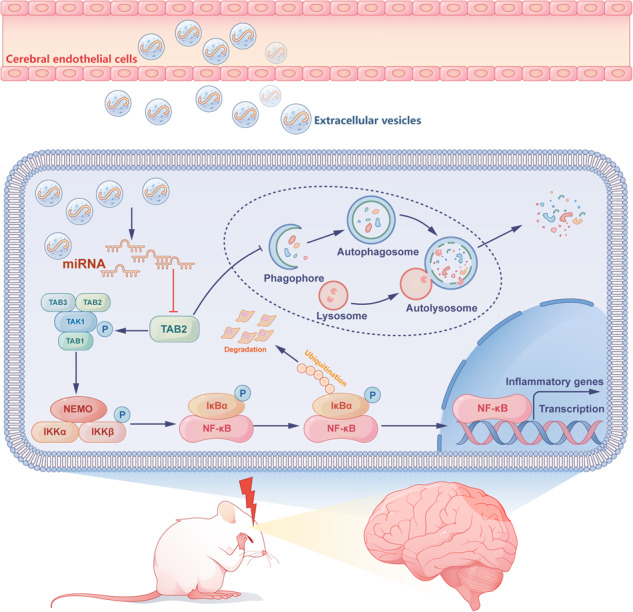

## Introduction

Chronic subclinical inflammatory status is one of the most well-established features shared by multiple neuropsychiatric and neurodegenerative disorders [1]. Sustained inflammatory activation would affect neurotransmission, neural apoptosis, and brain energy metabolism [2, 3]. Microglia, which are brain-resident macrophages, play a crucial role in innate immunity within the central nervous system [4, 5]. Prolonged stress triggers microglial activation, disrupting CNS homeostasis; they drive neuroinflammation via immune mediator secretion [6]. Of note, the conventional classification of microglial activation into M1- and M2-like states, is now considered overly simplistic [7]. Nevertheless, it continues to serves as a valuable framework for generating mechanistic and therapeutic hypotheses [8].

Previous researches suggested that neurovascular uncoupling is involved in the pathogenesis of neuroinflammation-associated neurological disorders [9]. The multicellular crosstalk between cerebral endothelial cells (CEC) and brain parenchymal cells, including neuron and glia cells, is critical to maintain neurovascular homeostasis [10]. Meanwhile, targeting the blood-brain microenvironment is indicated as a novel therapeutic strategy against neurological dysfunctions. Extracellular vesicles (EVs) constitute a comprehensive term that encompasses diverse subtypes of membranous structures released by cells. These subtypes include exosomes, microvesicles, microparticles, ectoderm, epithelium, apoptotic bodies, and numerous other variations [11]. After the release, EVs can be taken up by recipient cells, transferring their cargo, such as active proteins, RNA species and small molecules, and thereby orchestrate the dynamic intercellular communication [12]. Previous researches demonstrated that CEC-derived EVs (CEC-EVs) confer neuroprotective actions in the animal models of brain ischemia, stroke, and Alzheimer’s disease, preventing the brain from neuronal loss, oxidative damage and excessive neuroinflammation [13–16]. However, the molecular mechanisms underlying the neuroactivity remain still elusive.

Based on these clues, the present study aims to firstly investigate the role of CEC-EVs in the blood-brain communication under inflammatory condition. We assessed the effect of CEC-EVs on lipopolysaccharide (LPS)-induced inflammatory process and microglial polarization. Meanwhile, the impacts of inflammatory activation on CEC-EVs release and assimilation were also evaluated. To elucidate the mechanism, we then analyzed microRNA (miRNA) profile to explore the potential active molecular that mediated the anti-inflammatory action of CEC-EVs. Moreover, we also found that through inhibiting TGFβ-activated kinase 1 (TAK1)-binding proteins 2 (TAB2), miR-672-5p derived from CEC-EVs suppressed TAK1-TAB signaling and thereby mitigating the neuroinflammatory activation.

## Results

### Inflammatory activation had no effect on EVs release in CECs but increased microglial uptake of CEC-EVs

**Fig. 1:**
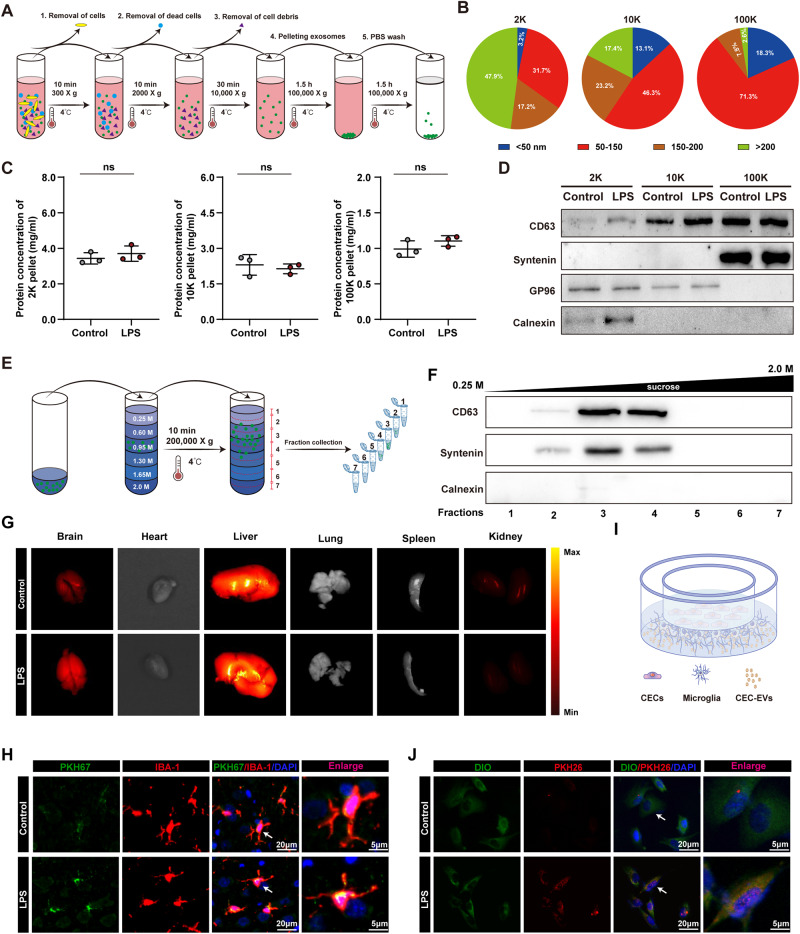
LPS treatment had no effect on EVs release in CECs but increased microglial uptake of CEC-EVs. **A** Schematic diagram of EVs isolation from conditioned media. **B** Size distribution of EVs in 2 K, 10 K and 100 K pellets. **C** Total protein concentrations of 2 K, 10 K and 100 K pellets derived from control group and LPS group. **D** 2 K, 10 K and 100 K pellets were blotted for the exosomal markers CD63 and syntenin, and for the endoplasmic reticulum marker GP96 and Calnexin. **E** Flow charts for the EVs purification procedure based on sucrose density gradient centrifugation. **F** Western blot for exosomal markers CD63, syntenin and Calnexin in different sucrose gradient fractions. **G** Representative imaging distribution of the DiR-labeled CEC-EVs in different organs, including brain, heart, liver, spleen, lung, and kidney. **H** CEC-EVs can cross the blood-brain barrier and neuroinflammatory markedly enhanced the incorporation of CEC-EVs into microglia. **I** Transwell co-culture system. **J** LPS treatment increased microglial uptake of CEC-EVs. The lipid membrane of CECs was stained with PKH26 red fluorescent dye and the cells were co-cultured with BV2 microglial cells stained with DIO in a Transwell. All data are present as means ± SD (*n* = 3). Unpaired Student’s *t*- test. ns not significant.

Ultracentrifugation is the standard EVs purification method [17]. We isolated CEC-derived EVs, comparing pellets from low (2 K), medium (10 K), and ultracentrifugation (100 K) speeds in control and LPS-treated cells (Fig. 1A). NTA data showed majority of 2 K pellets with >200 nm diameter, while 10 K and 100 K pellets had exosome-size distribution (50–150 nm) (Fig. 1B).

Protein concentrations were similar between LPS-treated and Control groups in 2 K, 10 K, and 100 K pellets (Fig. 1C). Western blot assessed EV markers in 2 K, 10 K, and 100 K pellets. CD63, syntenin enriched in 100 K pellets, while endoplasmic reticulum protein GP96, Calnexin were nearly undetectable (Fig. 1D), confirming successful small EV isolation. However, no notable differences were observed in LPS-treated vs. Control groups in 2 K, 10 K, 100 K pellets (Fig. 1D).

CEC-EVs were further purified using sucrose density gradient centrifugation (Fig. 1E). Immunoblotting of fractions (1–7) from the gradient revealed absence of calnexin signal, confirming no contamination of endoplasmic reticulum status, whereas CD63 and syntenin were enriched in fractions 2–4 (Fig. 1F). Fractions 3 and 4 were chosen for subsequent analysis.

Typically, sphere-shaped EVs with double-membrane structures were observed in both control and LPS treated group. No morphological difference of the EVs was observed between the two groups concerning the shape (Fig. S1A). The NTA data also demonstrated that EVs displayed a comparable size distribution, with most electric vehicles being distributed within the range of 50–150 nm, which coincides with the diameter of exosomes (Fig. S1B). The levels of EVs-specific markers, such as Alix and TSG101 in whole cell lysates (WCL) and EVs fractions were unchanged by LPS exposure (Fig. S1C). Calnexin was hardly detectable in the EVs fractions but was abundant in WCL, indicating successful EVs isolation (Fig. S1C).

To explore EVs distribution in vivo, DiR-labeled EVs were intravenously injected into mice. DiR signal appeared in brain, liver, and kidney tissues, implying multi-organ targeting by CEC-EVs, with intensified brain signal post-LPS treatment (Fig. 1G). We next explored EVs absorption by neural cells and neuroinflammatory influence (Fig. S1D). PKH67-labeled EVs were intravenously injected, revealing blood-brain barrier crossing and absorption by neurons, astrocytes, and microglia (Figs. S1E and 1H). Neuroinflammation augmented microglial incorporation of CEC-EVs (Fig. 1H). To validate in vivo findings, a Transwell co-culture (membrane pore = 0.4 μm) of CECs (PKH26 labeled) and BV2 microglia or primary microglia (DIO labeled) confirmed microglial uptake (Fig. 1I). LPS increased PKH26 transfer, indicating increased EVs uptake by activated microglia (Figs. 1J and S1F).

### CEC-EVs contains anti-inflammatory actions

**Fig. 2:**
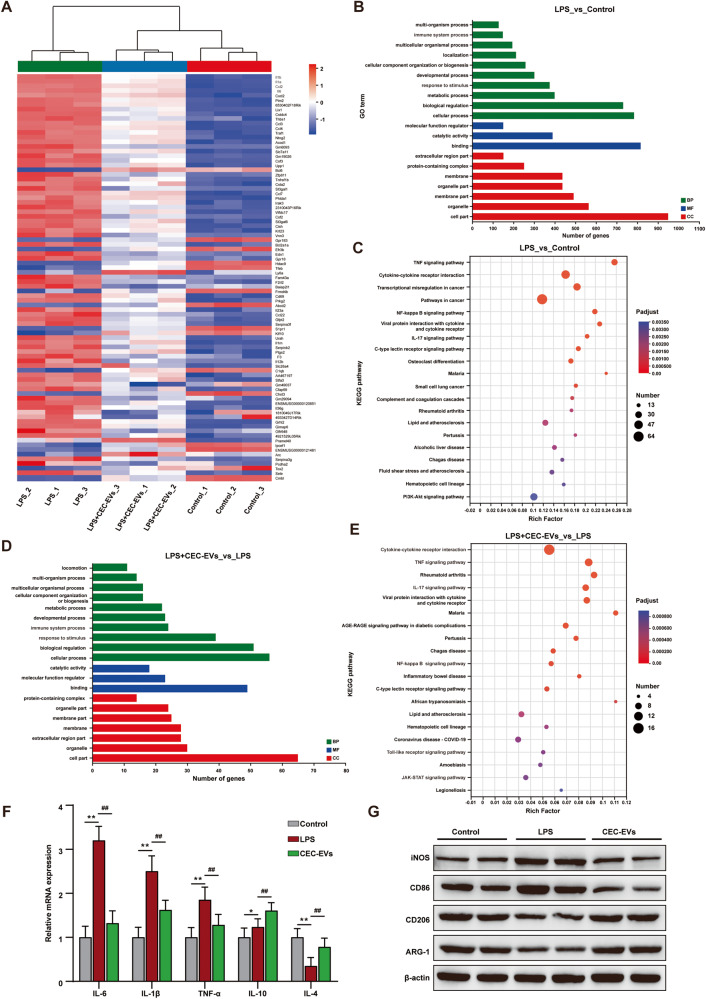
Effect of CEC-EVs on immune responses in microglia. **A** Heatmap visualization of the expression levels of the differentially expressed genes (DEGs). The colors indicate the expression level, ranging from red (high expression level) to blue (low expression level). The annotation of GO functions (**B**) and Kyoto Encyclopedia of Genes and Genome (KEGG) (**C**) pathway analysis of DEGs from microglia after LPS treatment. The annotation of GO functions (**D**) and KEGG (**E**) pathway analysis of DEGs from microglia after CEC-EVs treatment. **F** CEC-EVs alleviates LPS-induced microglial inflammatory responses. One-way ANOVA. **G** The expression of microglial pro-inflammatory marker (iNOS and CD86) and anti-inflammatory marker (CD206, ARG-1) were examined by western blot. All data are present as means ± SD (*n* = 3). *p < 0.05, **p < 0.01 compared to control group. ^##^*p* < 0.01 compared to LPS group.

Considering CEC-EVs’ neuroprotective actions, we performed transcriptomics to assess their neuroimmune-modulatory effects. We identified 27163 genes, with Q30 bases exceeding 94.63%, indicating high data quality. Error rate (Fig. S2A), base content (Fig. S2B), saturation curve (Fig. S2C), and gene expression dispersion (Fig. S2D) confirmed sequencing soundness. Hierarchical clustering revealed closer similarity between LPS + CEC-EVs and control groups than LPS group (Fig. 2A). Volcano plots demonstrated significant changes in pro-inflammatory cytokines and chemokines, such as IL-6, TNF-α, and CCL2 (Fig. S2E, F). Gene ontology (GO) and KEGG enrichment analysis were performed to categorize the differentially expressed genes (DEGs). LPS treatment mainly enriched immune system and response to stimulus (Fig. 2B). KEGG identified pathways like TNF, NF-kappa B, and IL-17 signaling pathways (Fig. 2C). CEC-EVs treatment showed similar GO results (Fig. 2D), impacting TNF, NF-kappa B, IL-17, and Toll-like receptor pathways (Fig. 2E). Toll-like receptor signaling and NF-kappa B are innate immunity and inflammation keys, activating inflammatory factors’ release, regulating responses [18, 19].

Our data also showed that after LPS stimuli, the microglial cells were prone to be induced release inflammatory cytokines and inhibited the expression of anti-inflammatory factors (Fig. 2F). As expected, the CEC-EVs can ameliorate LPS-induced overactivation directly (Fig. S2G). We also found that CEC-EVs shifted the microglial polarization from pro-inflammatory phenotype to anti-inflammatory phenotype, increasing the biomarkers of anti-inflammatory phenotype but decreasing the pro-inflammatory mediator secretion (Fig. 2G). The immunofluorescence results also confirmed this finding and demonstrated that the increased the abundance of iNOS and decreased ARG-1 induced by LPS exposure were markedly mitigated by CEC-EVs treatment, highlighting the robust anti-inflammatory actions of CEC-EVs (Fig. S2H). These comprehensive data suggest that CEC-EVs alleviated LPS-induced inflammatory response and shifted the microglial polarization.

### miR-672-5p is transferred from CECs to microglia via EVs-mediated delivering mechanism

**Fig. 3:**
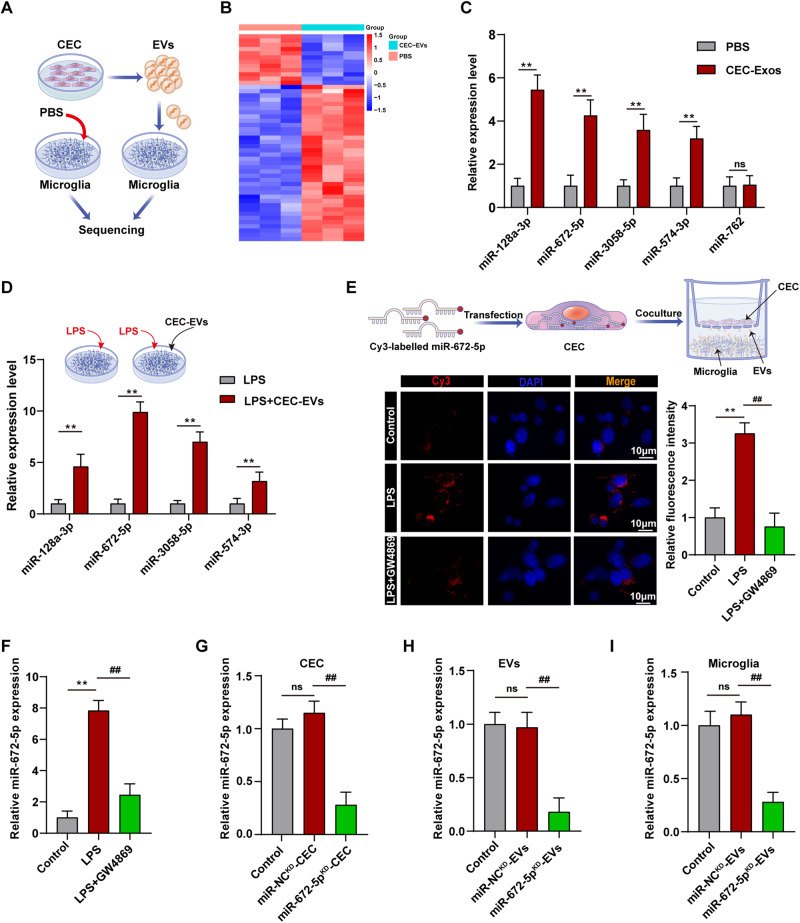
miR-672-5p was transferred from CECs to microglia. **A** The schematic diagram of microglia sequencing analysis after CEC-EVs treatment. **B** The heat map plot of the upregulated and downregulated miRNAs between microglia treated with CEC-EVs and PBS. **C** Validation of upregulated genes by qRT-PCR. Unpaired Student’s *t* test. **D** qRT-PCR was used to explore the expression of miRNAs including miR-128-3p, miR-672-5p, miR-3058-5p, miR-574-3p and miR-762 in activated microglia treated with EVs. Unpaired Student’s *t* test. **E** The EVs secretion inhibitor, GW4869, inhibited miR-672-5p transfer from CECs to microglia. one-way ANOVA. **F** Inhibition effect of GW4869 on miR-672-5p transfer was determined by qRT-PCR. One-way ANOVA. **G** miR-672-5p knockdown in CECs and the knockdown efficiency was confirmed by qRT-PCR, one-way ANOVA. Effect of miR-672-5p knockdown in CECs on miR-672-5p expression in CEC-EVs (**H**) and microglia (**I**), respectively, one-way ANOVA. All data are present as means ± SD (*n* = 3). ns not significant, **p < 0.01 compared to control group. ^##^*p* < 0.01 compared to miR-NC^KD^-CEC or miR-NC^KD^-EVs group.

miRNAs are the major cargo of EVs and play an essential role in the communication between cells. To investigate the potential immune-modulatory mechanism, we isolated RNA specifically from CEC-EVs- or PBS-treated microglia to perform miRNA sequencing analysis (Fig. 3A). The results showed that CEC-EVs increased 37 miRNAs (>1.5-fold, *p* < 0.05) and decreased 12 miRNAs (Fig. 3B). Following miRNA profiling, top five upregulated miRNAs, including miR-128-3p, miR-672-5p, miR-3058-5p, miR-574-3p and miR-762 were selected to be validated by q-PCR (Fig. 3C). As expected, CEC-EVs increased these miRNAs in LPS-treated microglia. Particularly, miR-672-5p displayed a more pronounced increase in response to CEC-EVs under LPS stimulation (Fig. 3D). This finding was correlated with the enhanced CEC-EVs uptake upon LPS exposure. To further confirm the EVs-mediated miR-672-5p transporting mechanism, Cy3-labeled miR-672-5p mimics were transfected into CECs and co-cultured with microglial cells in Transwell plates to track miR-672-5p intercellular movement. Cy3-labeled miR-672-5p appearance in microglial cells indicated successful transfer from upper chamber CECs to lower chamber microglia, which was blocked by GW4869, an EV secretion inhibitor, supporting that miR-672-5p is transported between cells through EVs (Fig. 3E). Also, the results of the miR-672-5p transport were verified by qPCR (Fig. 3F). Migration is integral to microglial responses to inflammation, prompting exploration into miR-672-5p’s role in microglial migration and inflammation regulation [20]. Migration assays revealed that miR-672-5p mimic inhibited microglial migration (Fig. S3A) and enhanced anti-inflammatory action (Fig. S3B–E). Next, we successfully knockdown the miR-672-5p in CECs (Fig. 3G). The relative expression of miR-672-5p was validated in EVs isolated from miR-672-5p-NC^KD^-CECs and miR-672-5p^KD^-CECs, respectively (Fig. 3H). Correspondingly, miR-672-5p^KD^-EVs treated microglia showed a dramatic decrease of miR-672-5p expression compared with miR-NC^KD^-EVs treated group (Fig. 3I).

### miR-672-5p suppressed the expression of TAB2 and inhibited the interaction between TAB2 and TAK1

**Fig. 4:**
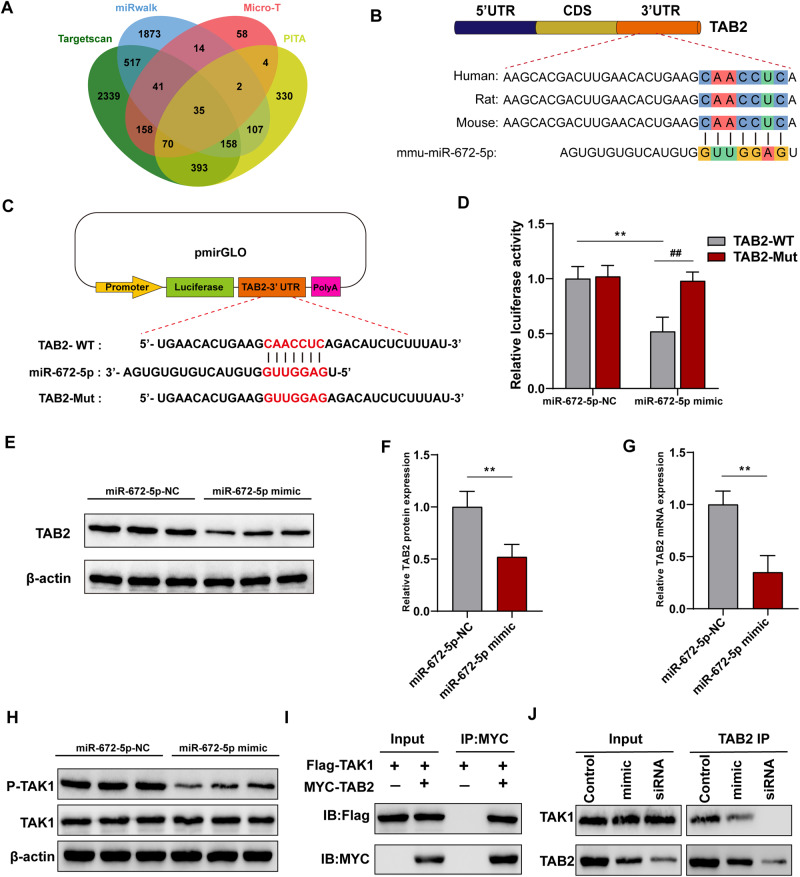
miR-672-5p negatively regulated TAB2 expression and inhibited the interaction between TAB2 and TAK1. **A** Candidate gene targets were predicted by intersecting outputs from four distinct prediction software programs (TargetScan, miRWalk, micro-T, and PITA). **B** miR-672-5p targets the 3′-UTR of TAB2 with a high conservation. **C**, **D** Luciferase activity of the wild-type (TAB2-WT) or mutant (TAB2-Mut) TAB2 3’UTR reporter gene transfected with the miR-672-5p mimics or miR-672-5p-NC (negative control). one-way ANOVA. Effect of the transfection of miR-672-5p mimics on expression level of TAB2 protein (**E**, **F**) and mRNA (**G**). Unpaired Student’s *t* test. **H** Effect of the transfection of miR-672-5p mimics on protein expression level of TAK1 and its phosphorylation. **I** Co-immunoprecipitation (Co-IP) experiments were performed with anti-MYC antibody on microglia cell expressing Flag-TAK1 and MYC-TAB2. **J** Transfection of miR-672-5p mimics or knockdown TAB2 attenuated the interaction between TAB2 and TAK1. All data are present as means ± SD (*n* = 3). **p < 0.01 compared to TAB2-WT in miR-672-5p-NC treatment group or miR-672-5p-NC group. ^##^*p* < 0.01 compared to TAB2-WT group in miR-672-5p mimic treatment group.

To identify the downstream target of miR-672-5p, we predicted intersected target genes from four databases (Fig. 4A). Of these potential target genes, 3’-UTR of TAB2, which is tightly related to inflammation, was found to bound to miR-672-5p with a high affinity and high conservation (Fig. 4B). TAB2 is an indispensable co-activator of TAK1 signaling, which can be activated by inflammatory stimuli, such as TNFα, IL-1β and toll-like receptor ligands. Then, the TAK1-TAB complex phosphorylates IKK, which further triggers NFκB activation by inducing IκB phosphorylation and degradation, thereby facilitating inflammatory process. To further confirm the 3’-UTR of TAB2 is a direct target of miR-672-5p, we constructed TAB2 3’UTR wild-type (TAB2-WT) or mutated (TAB2-Mut) plasmids in the pmirGLO dual-luciferase reporter vector (Promega), and luciferase activity was measured after co-transfection of miRNA and luciferase plasmids (Fig. 4C). We found that miR-672-5p mimic significantly reduced the luciferase activity of TAB2-WT compared with mimic-NC, with no significant change in TAB2-Mut, confirming the target binding of miR-672-5p (Fig. 4D). Additionally, miR-672-5p mimic also suppressed TAB2 expression at protein and mRNA levels (Fig. 4E–G). inhibiting TAK1 phosphorylation but not protein level (Fig. 4H). TAB2 forms a complex with TAK1 and TAB1 [21], and Co-immunoprecipitation (Co-IP) confirmed their interaction in microglial cells (Fig. 4I), reduced upon TAB2 knockdown (Fig. 4J).

### CEC-EVs-derived miR-672-5p suppressed pro-inflammatory NFκB activation by targeting TAB2

**Fig. 5:**
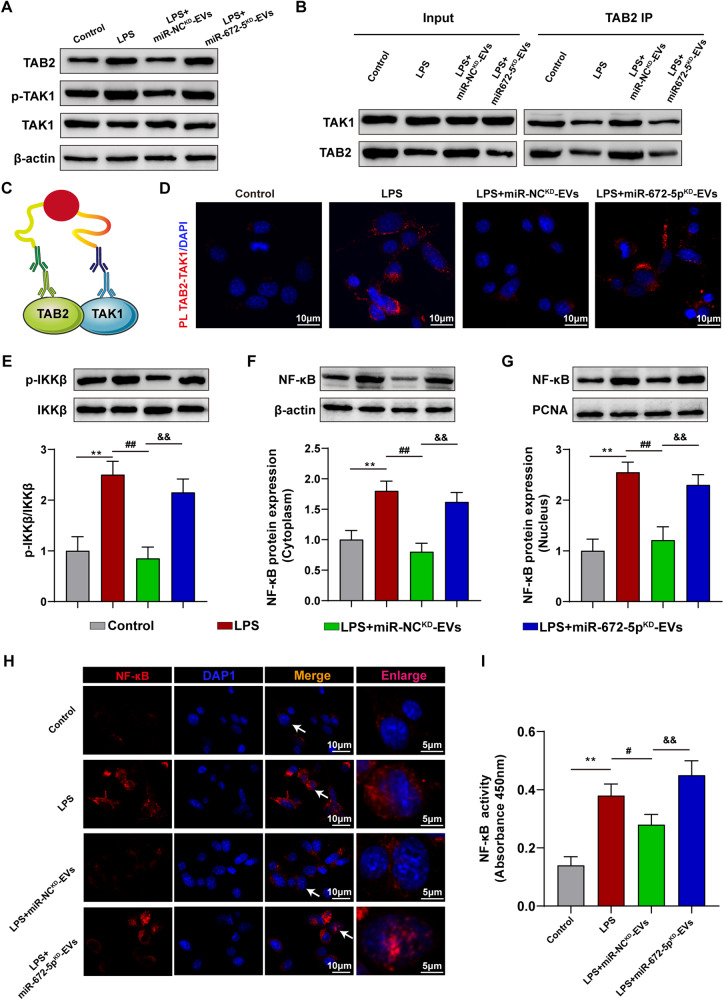
CEC-EVs-derived miR-672-5p regulated interaction between TAB2 and TAK1 and suppressed NFκB activation in microglia. **A** Effect of the CEC-EVs-derived miR-672-5p on protein expression level of TAB2, TAK1 and p-TAK1. **B** CEC-EVs-derived miR-672-5p attenuated the interaction between TAB2 and TAK1. **C** Schematic diagram of the of in situ proximity ligation assay (PLA) in microglia cells of TAB2 and TAK1. **D** PLA confirms that CEC-EVs-derived miR-672-5p inhibited the interaction between TAB2 and TAK1. CEC-EVs-derived miR-672-5p promoted the protein expression of p-IKKβ (**E**) and NFκB (**F**, **G**) nuclear translocation. **H** Representative images of immunofluorescence assays of NFκB in vitro. **I** CEC-EVs-derived miR-672-5p suppressed the activity of NFκB. All data are present as means ± SD (*n* = 3). One-way ANOVA. **p < 0.01 compared to Control group. ^#^*p* < 0.05, ^##^*p* < 0.01 compared to LPS group. ^&&^*p* < 0.01 compared to LPS + miR-NC^KD^-EVs group.

Considering that the miR-672-5p could negatively regulate TAB2 expression, we examined CEC-EVs’ miR-672-5p impact on TAB2 and TAK1 expression. miR-NC^KD^-EVs attenuated LPS-induced TAB2 and p-TAK1 upregulation, reversed by miR-672-5p^KD^-EVs (Fig. 5A). TAK1 levels remained unchanged with miR-NC^KD^-EVs or miR-672-5p^KD^-EVs (Fig. 5A). Co-IP data showed miR-NC^KD^-EVs reduced TAB2-TAK1 interaction (Fig. 5B), confirmed by PLA (Fig. 5C, D). NF-κB plays important roles in immune and inflammatory responses, and the activated TAB2-TAK1 complex can phosphorylate the IκB kinase (IKK) complex, further activating NF-κB [22, 23]. Hence, we investigated whether CEC-EVs affected the activation of NF-kB. Notably, miR-NC^KD^-EVs ameliorated LPS-induced IKKβ phosphorylation (Fig. 5E), IκB degradation and NF-κB activation, the process of which was also reduced by miR-672-5p^KD^-EVs knockdown in CEC-EVs (Fig. S4A, B). Nuclear translocation of NF-κB revealed that miR-NC^KD-^EVs decreased NF-κB translocation from cytoplasm to nucleus (Fig. 5F–H). Similarly, NFκB activity is also inhibited by miR-NC^KD^-EVs treatment (Fig. 5I). We also found that the miR-NC^KD-^EVs inhibited microglial migration, while miR-672-5p^KD^-EVs promoted cell migration (Fig. S4C). Moreover, miR-NC^KD-^EVs inhibited inflammatory cytokines produced by activated microglia (Fig. S4D–G).

**Fig. 6:**
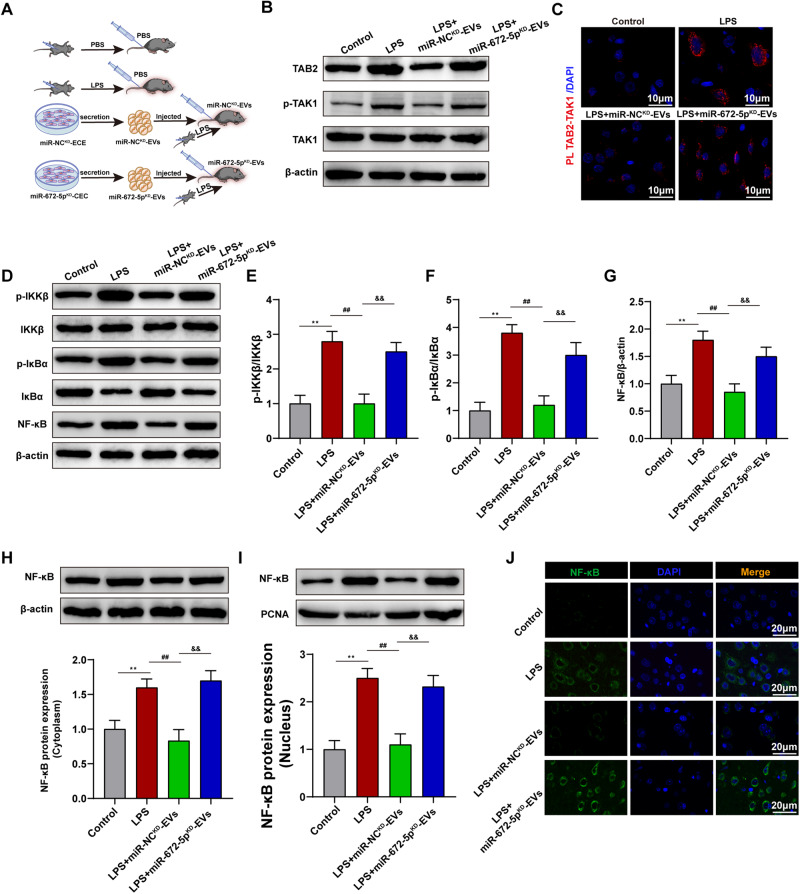
CEC-EVs-derived miR-672-5p regulated interaction between TAB2 and TAK1 and suppressed NFκB activation in vivo. **A** Schematic diagram of the of CEC-EVs in vivo experimental study. **B** Effect of the CEC-EVs-derived miR-672-5p on protein expression level of TAB2, TAK1 and p-TAK1 in vivo. **C** PLA confirms that CEC-EVs-derived miR-672-5p inhibited the interaction between TAB2 and TAK1. **D** Representative western blots of p-IKKβ, IKKβ, p-IκBα, IκBα, NFκB. Statistical graphs of protein expression of p-IKKβ (**E**), p-IκBα (**F**), NFκB (**G**). **H**, **I** CEC-EVs-derived miR-672-5p promoted NFκB nuclear translocation. **J** Representative images of immunofluorescence assays of NFκB in vivo. All data are present as means ± SD (*n* = 6). One-way ANOVA. **p < 0.01 compared to Control group. ^##^*p* < 0.01 compared to LPS group. ^&&^*p* < 0.01 compared to LPS + miR-NC^KD^-EVs group.

To further explore the mechanism of CEC-EVs-derived miR-672-5p action in vivo, miR-NC^KD^-EVs or miR-672-5p^KD^-EVs were injected into mice via tail veins (Fig. 6A). miR-NC^KD^-EVs suppressed LPS-induced TAB2 expression and TAK1 activation, counteracted by miR-672-5p^KD^-EVs (Fig. 6B). In accordance, miR-NC^KD^-EVs weakened TAB2-TAK1 interaction (Fig. 6C). In this light, NF-κB activation was further explored in vivo, revealing miR-NC^KD^-EVs suppressing phosphorylation of IKKβ, IκBα, and NF-κB activation (Fig. 6D–G). miR-NC^KD^-EVs attenuated LPS-induced NF-κB nuclear translocation (Fig. 6H, I), confirmed by immunofluorescence (Fig. 6J). These data highlight CEC-EVs’ immune-regulatory role through miR-672-5p delivery.

### CEC-EVs-derived miR-672-5p promoted autophagy and suppressed inflammasome activation by targeting TAB2

**Fig. 7:**
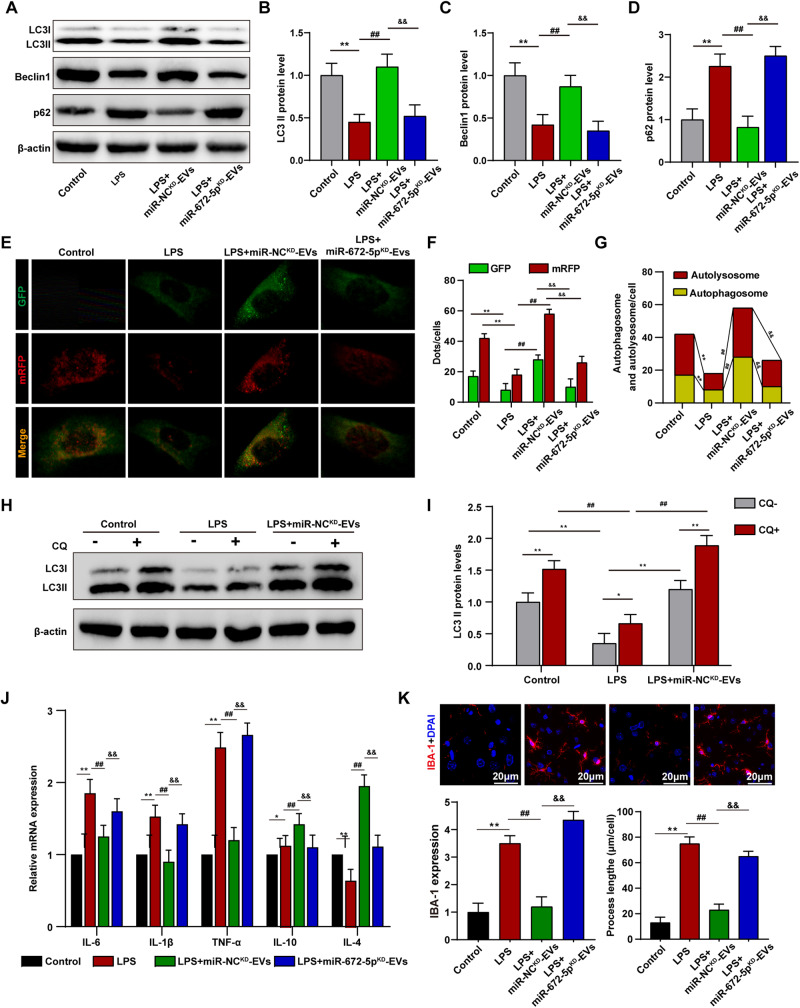
CEC-EVs-derived miR-672-5p promoted autophagy and suppressed NLRP3 inflammasome activation. **A** CEC-EVs-derived miR-672-5p enhances LC3II, Beclin1 level and decreases SQSTM1/p62 expression. **B**–**D** Statistical graphs of protein expression of LC3II, Beclin1 and SQSTM1/p62. **E** Immunofluorescence staining of microglial cells transfected with mRFP-GFP-LC3 adenovirus. **F**, **G** Calculated numbers of autophagosome (GFP^+^ RFP^+^ yellow puncta) and autolysosome (red puncta) numbers. All data are present as means ± SD (*n* = 3). One-way ANOVA. **p < 0.01 compared to Control group. ^##^*p* < 0.01 compared to LPS group. ^&&^*p* < 0.01 compared to LPS + miR-NC^KD^-EVs group. **H**, **I** Chloroquine (CQ) was used to evaluate the effects of CEC-EVs treatment or LPS treatment on LC3II. CEC-EVs-derived miR-672-5p alleviates LPS-induced microglial inflammatory responses (**J**) and activation (**K**). All data are present as means ± SD (*n* = 6). One-way ANOVA. *p < 0.05, **p < 0.01 compared to CQ− group. ^##^*p* < 0.01 compared to CQ+ group.

Except for NFκB pathway, TAB2 may interact with Beclin-1 to blunts autophagic process. Autophagy also restrains excessive inflammatory responses by degrading pro-inflammatory stimuli such as cytokines and inflammasomes. Therefore, we further assessed the impacts of CEC-EVs on autophagy. While LPS inhibits autophagy, miR-NC^KD^-EVs not only increased LC3II and Beclin-1 levels but also decreased SQSTM1/p62 expression (Fig. 7A–D), with the elevated LC3 II and Beclin-1 being further confirmed through immunostaining (Fig. S5A). Autophagic flux is generally referred to as a means to measure of autophagic degradation [24]. Once the autophagosome is fused to the lysosome, the GFP signal is quenched by the acidic environment and only RFP signal is observed [25]. LPS significantly decreased both the yellow and red puncta, whereas miR-NC^KD^-EVs facilitated autophagic flux (Fig. 7E–G). It is well known that autophagy is tightly associated with inflammation [26]. We then examined if CEC-EVs-regulated autophagy affected NLRP3 inflammasome in microglia. miR-NC^KD^-EVs enhanced autophagy, reduced NLRP3 inflammasome (Fig. S5B), suppressing NLRP3 activation (Fig. S5C, D). These findings all indicated that CEC-EVs may confer immune-regulatory and neuroprotective effects though miR-672-5p-TAB2 signaling. TNF-α, IL-1β, IL-6, IL-10 expression was also confirmed this point (Fig. S5E–H).

To grasp CEC-EVs-derived miR-672-5p and autophagy in vivo, we evaluated autophagy-related proteins. CEC-EVs restored autophagic markers downregulated by LPS exposure, absent in miR-672-5pKD-EVs (Fig. S6A–D). We further confirmed the role of CEC-EVs in autophagy by using chloroquine (CQ), an autophagy inhibitor. CQ induced the additional increase of LC3-II protein, whereas miR-NC^KD^-EVs treatment significantly improved the LPS-induced decrease of LC3-II expression (Fig. 7H, I). These results together suggested that LPS-induced autophagy dysfunction was mitigated by miR-NC^KD^-EVs. NLRP3 inflammasome protein expressions matched in vitro results (Fig. S6E, F). We then explored the immune-regulatory function of CEC-EVs in vivo, showing miR-NC^KD^-EVs alleviated LPS-induced neuroinflammation (Fig. 7J, K), shifting the microglial cells to anti-inflammatory phenotype (Fig. S6G, H).

### CEC-EVs improved LPS-induced behavioral deficits and neuroinflammation in mice

**Fig. 8:**
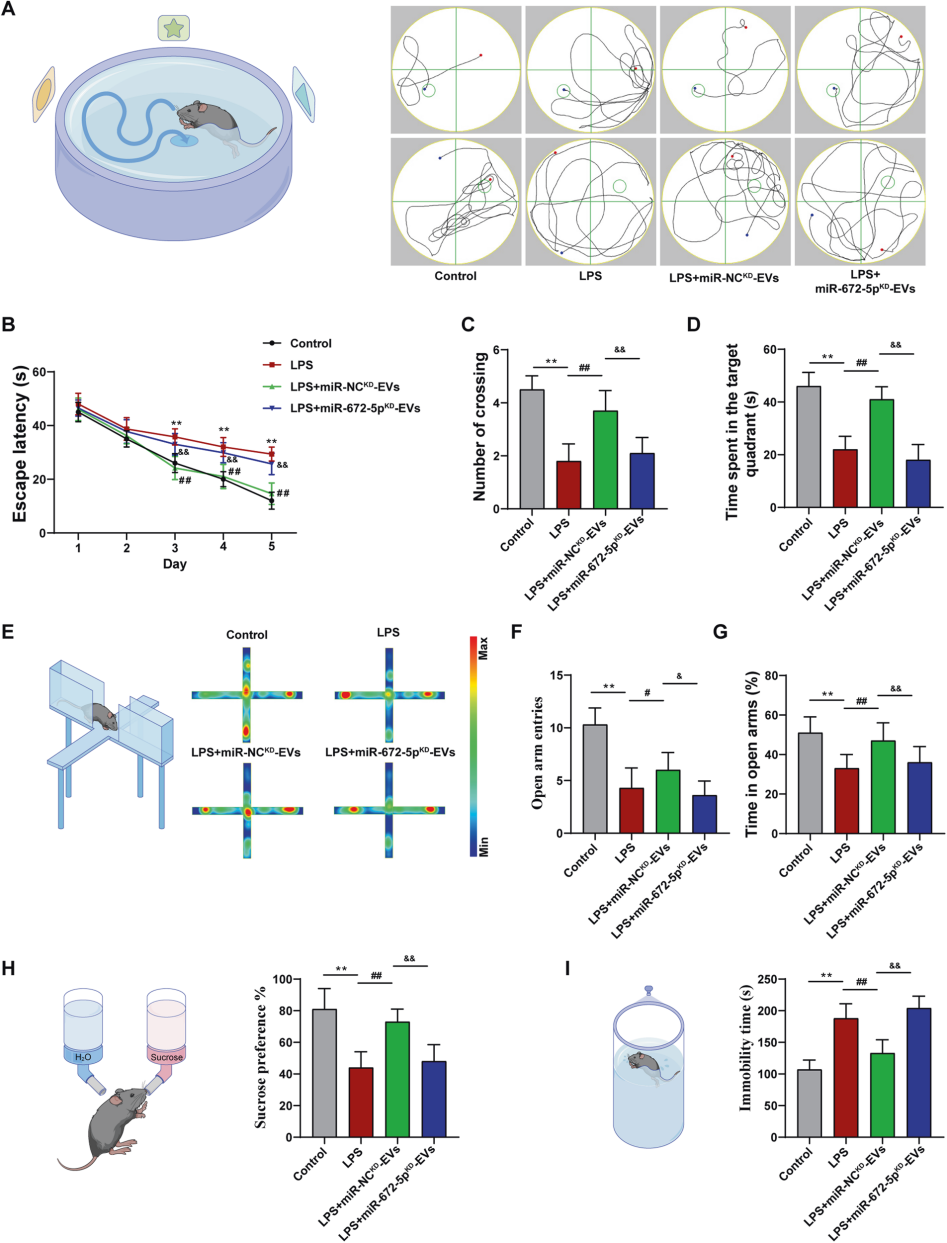
The neuroprotective effects of CEC-EVs in LPS-induced behavioral deficits in mice. **A** Representative traces indicating the paths of the mice in the space exploration and orientation navigation test. **B** CEC-EVs treatment reduced the time spent to find the submerged platform. CEC-EVs treatment enhanced the number of crossing (**C**) and reduced the time spent on exploring the quadrant that initially contained the platform (**D**). **E** Representative heat maps showing EPM results. CEC-EVs treatment increased number of open arm entries (**F**) and time spent in the open arm of the EPM test (**G**). **H** CEC-EVs treatment increased sucrose preference in the SPT. **I** CEC-EVs treatment decreased immobility time in the FST. All data are present as means ± SD (*n* = 12). One-way ANOVA. **p < 0.01 compared to Control group. ^#^*p* < 0.05, ^##^*p* < 0.01 compared to LPS group. ^&^*p* < 0.05, ^&&^*p* < 0.01 compared to LPS + miR-NC^KD^-EVs group.

To confirm the neuroprotective effects of CEC-EVs in vivo, CEC-EVs was administrated following repeated LPS stimuli. CEC-EVs treatment mitigated LPS-induced cognitive decline, evidenced by reduced escape latency in the learning curve and improved probe test performance (Fig. 8A–D). Additionally, CEC-EVs ameliorated LPS-induced anxiety-like and depression-like state. In the elevated plus maze (EPM) test, LPS exposure decreased the time spent in the open arm and the number of open arm entries, which was restored by CEC-EVs administration (Fig. 8E–G). Likewise, CEC-EVs also increased the sucrose preference in sucrose preference test (SPT) (Fig. 8H) and decreased the immobility time in forced swim test (FST) (Fig. 8I). In parallel, CEC-EVs treatment remitted LPS-induced neuronal apoptosis and neuronal loss, in Nissl staining and Tunel staining respectively, and the neuroprotective action of CEC-EVs was also abrogated by miR-672-5p inhibition (Fig. S7A–D).

## Discussion

There is accumulating evidence suggesting that EVs are critical in the maintenance of brain cell–cell communication and homeostasis [27]. In recent years, EVs have been separated from almost every type of neural cells, including neuron, astrocyte, and microglia, but there only few studies focusing on the activity of EVs derived from brain endothelial cells. Considering that the cerebrovascular system contributes to the contact between brain and periphery, the CEC-EVs are very likely to have a major role in the intercellular communication in the neurovascular unit. Therefore, the present study aims to investigate the function of CEC-EVs, especially in the context of inflammation.

Although previous researches showed that CEC-EVs contain neuroprotective activities, the molecular mechanism is still unknown. The present study firstly examined the role of CEC-EVs in the conditions of repeated inflammatory stimuli. We found that LPS had no effect on CEC-EVs release, whereas the ability of uptaking CEC-EVs was enhanced in the activated microglia following LPS exposure both in vitro and in vivo. Given the LPS-induced microglial activation, our study suggested that the pro-inflammatory phenotype of microglia was more prone to absorb CEC-EVs. This finding is in line with previous research that more EVs were accumulated in the pro-inflammatory type of macrophage in the lymph nodes, which might be attributed to the enhanced ability of phagocytosis [28]. Moreover, recent research showed that the serum-derived EVs are mostly uptaken by recipient mouse microglia after exposure to LPS [29]. The enhanced uptaken of CEC-EVs tends to constitute a compensatory mechanism of microglia in response to the sustained inflammatory stimuli. Meanwhile, we also found the anti-inflammatory effects of CEC-EVs, decreasing the expression of pro-inflammatory cytokines or mediators secreted by pro-inflammatory microglia but increasing the biomarkers of pro-inflammatory phenotype. These results imply that the neuroimmune-modulatory function of CEC-EVs, at least partly, contribute to its neuroprotective effects that found in stroke, ischemia/reperfusion injury and Alzheimer’s disease [14, 15].

As miRNA is one kind of the major properties that EVs transport [29], we move forward to examine the potential active miRNA delivered by CEC-EVs to microglia by using miRNA sequencing. We found that miR-128a-3p and miR-672-5p were increased by CEC-EVs treatment, and particularly, miR-672-5p was increased to a greater extent than miR-128a-3p following CEC-EVs treatment in LPS treatment microglia. These results were in line with the LPS-induced CEC-EVs uptaken. We then confirmed miR-672-5p was transferred by CEC-EVs by using Cy3-labeled miR-672-5p mimics, and selectively knocking down its expression in CEC-EVs markedly suppressed CEC-EVs-induced miR-672-5p expression in microglial cells. The direct anti-inflammatory action of miR-672-5p was also proved by us, suggesting that the miR-672-5p mediates the immune-regulatory function of CEC-EVs [30].

Bioinformatic analysis was then performed and we found that TAB2 is an interesting target of the intersecting outputs from four predicting programs. Given that TAB2 is tightly related to inflammatory signaling, particularly NFκB activation, we then showed that miR-672-5p can bind with 3’UTR of TAB2 and negatively regulated its expression by using luciferase reporter assay and western blot, respectively. TAB2 is the co-activator of TAK1, whose activation triggers NFκB translocation and induces the expression of pro-inflammatory cytokines [23, 31]. The deficiency of TAB2 would impair the activation of TAK1 and NFκB signaling, reducing the transcription of downstream genes 31. Meanwhile, suppressing TAB2 also undermined LPS-induced microglial activation and targeting TAB2 have been demonstrated as an underlying drug target to ameliorate inflammation-induced tissue damage [32, 33]. Our data are in line with these results and shows that CEC-EVs reduced TAB2, suppressing LPS-induced TAK1-NFκB signaling, whereas reduction of miR-672-5p expression abrogated CEC-EVs anti-inflammatory action.

Notably, TAB2 may interact with Beclin-1 to inhibit autophagy, a process that responsible for the selective degradation of damaged organelles and targeted proteins [34, 35]. Through catabolizing inflammatory cytokines or other mediators, such as inflammasomes, autophagic process maintains homeostasis to avoid excessive and prolonged inflammation-induced injury [36, 37]. Therefore, the impact of CEC-EVs on autophagy was also investigate. As we and other researchers previously reported, LPS exposure suppressed autophagy and increased the expression of TAB2 [38, 39]. Although previous research suggested that Forkhead box O3 (FOXO3) is involved in the suppressed autophagy following LPS-induced TLR4 activation, our data demonstrated that the elevated expression of TAB2 enhanced its interaction with Beclin1, thereby, at least partially, contributing to the autophagic inhibition [40]. The impaired autophagy may lead to the pro-inflammatory mediators accumulation and accelerate the inflammation-induced damage, whereas CEC-EVs treatment reduced the interaction between TAB2 and Beclin1 and facilitated autophagic flux. Therefore, TAB2 is a key common target shared by NFκB signaling and autophagic process. On the other hand, CEC-EVs may contain immune-regulatory and neuroprotective action via inhibiting TAB2 expression.

To test the hypothesis in vivo, CEC-EVs were intravenously injected following repeated LPS exposure. Chronic subclinical neuroinflammation is associated with various neuropsychiatric disorders, such as cognitive decline and depression [41]. Similar to our previous findings, sustained inflammatory stimuli caused cognitive decline, depression-like and anxiety-like behaviors, which was partly restored by CEC-EVs treatment. In correspondence, CEC-EVs administration restored the inflammatory status, shifting the microglia from pro-inflammatory phenotype to immunoregulatory pro-inflammatory phenotype and protecting the brain from LPS-induced deteriorating effects. In line with the results in vitro, CEC-EVs treatment also reduced brain TAB2 expression, restrained LPS-induced NFκB signaling and restored the autophagic flux. However, these actions were markedly compromised in miR-672-5p^KD^-EVs treated group, lending more weight to the theory that miR-672-5p is the active cargo transferred by CEC-EVs to exert the anti-inflammatory activity.

While our preliminary findings are promising, several potential limitations warrant further consideration. First, the dynamic nature of microglia in the intricate in vivo environment poses a challenge; in vitro cultured microglia might not faithfully replicate the true in vivo state [5, 42–44]. Second, microglial heterogeneity raises the need to ascertain if all microglia can uniformly uptake miR-672-5p [45, 46]. Third, compared to microglia that did not receive miR-672-5p, the detailed changes in intracellular signaling remain unexplored. Lastly, microglia subpopulations across brain or spinal cord regions necessitate future validation the role of miR-672-5p in these areas.

Collectively, our data demonstrated the anti-inflammatory action of CEC-EVs, which also significantly protected the bran from inflammation-induced nerve damage and behavioral deficits. We further showed that miR-672-5p derived from CEC-EVs suppressed TAB2 expression, thereby inhibiting NFκB signaling and facilitating autophagic process. the present study shed novel light into the cell–cell communication in the neurovascular unit, raising the possibility that CEC-EVs might be a novel treatment strategy in the neuropsychiatric disorders that associated with neuroinflammation.

## Materials and methods

### Animals and treatment

Male C57BL/6J mice aged 7–8 weeks were purchased from Jinan PengYue Laboratory Animal Breeding Co., Ltd (Jinan, China). The mice were housed under standard conditions (temperature: 22 ± 2 °C; 12:12 h light/dark cycle; humidity: 50 ± 5%) with free access to sterile food and water. The mice were habituated their environment for 7 days before the experiment. The mice were randomly divided into control, LPS, LPS + miR-NC^KD^-EVs and LPS + miR-672-5p^KD^-EVs group. In the LPS + miR-NC^KD^-EVs and LPS + miR-672-5p^KD^-EVs, 4 × 10^10^ particles were injected into mice via the tail vein 3 times per week for 2 weeks as previously reported [47], whereas the control and LPS groups were injected with the same volume of PBS via the tail vein. At the same time, based on our previous study [48], each mouse was received LPS via intraperitoneal injection at a dose of 500 μg/kg every 2 days for a total of seven injections. Twenty-four hours after the last injection, the behavioral studies were conducted over the next 3 days. After the final behavioral test, mice were deeply anesthetized with intraperitoneal injection of sodium pentobarbital (50 mg/kg body weight). Subsequently, the tissues were promptly removed and rapidly dissected on the ice surface. All animal procedures were conducted in accordance with Guide for the Care and Use of Laboratory Animals and were approved by the Ethics Committee of Jining First People’s Hospital (Approval number JNMC-2022-DW-041).

### Cells culture and treatment

Primary microglial cultures were prepared from the brains of C56BL/6J mice (postnatal day 1–2). Mouse brains were dissociated in culture medium containing DMEM/F12 supplemented with 10% fetal bovine serum (FBS, Gibco) by using pipettes. The cell suspension was then filtered through a 40-μm cell strainer to remove tissue debris. The cell suspension was plated in poly-D-lysine-coated T-75 flasks and incubated at 37 °C with 5% CO_2_. The culture medium was replaced with fresh culture media 3 days later. After 10–14 days, astrocytes were layered at the bottom of the T-75 flask, and microglia grew on top of the astrocyte layer. Microglia were then separated from mixed glial culture by gentle shaking for 30 min in an orbital shaker for further experiments.

Primary cerebral endothelial cells were isolated from adult mice according to previously protocols [49]. In brief, cerebral cortices was separated from brain and minced by sterile scissors in a petri dish filled with cold D-Hank’s solution. Minced tissue was then digested with 0.1% collagenase type II and dispase at 37 °C for 30 min. The endothelial cell fragments were separated with a 50% Percoll gradient. The endothelial cells were cultured in complete DMEM, which was supplemented with 20% FBS, 1% penicillin/streptomycin (Gibco) and 1% endothelial cell growth supplement (ScienCell). Cells were incubated at 37 °C in a 5% CO_2_ incubator. After cells reached 80% confluence, the medium was changed to DMEM supplemented with Exosome-free FBS medium (BS-1205, Inner Mongolia Opcel Biotechnology Co., Ltd). BV2 murine microglia cell line and bEnd.3 cell line were purchased from Procell Life Science & Technology Co., Ltd (Wuhan, China) and cultured as previous described [50]. BV2 cells and bEnd.3 cells were cultured in DMEM medium supplemented with 10% FBS and 1% penicillin/streptomycin (Gibco, Shanghai). Then, the cells were incubated at 37 °C in a 5% CO_2_ incubator and medium were changed every 2 days.

### EVs isolation and purification

EVs were isolated and purified from cell culture supernatants as previously described [51]. In total, 5–6 × 10^6^ cells were cultured per T75 culture flask (total eight flasks), each containing 10 ml exosome-free medium media. After 48 h, the cultured supernatant was collected and centrifuged at 300 × *g* for 10 min to remove cell contamination. Collecting supernatant and centrifuged at 2000 × *g* for 20 min (2 K pellet), followed by another centrifugation at 10,000 × *g* for 60 min (10 K pellet) to remove any possible dead cells and cell debris. Following centrifugation, the supernatant was collected and passed through a 0.22-μm sterile filter. The resulting supernatant were transferred to high-speed centrifuge Tubes (3139-0050, Thermo) and ultra-centrifugated at 100,000 × *g* for 90 min (100 K pellet). The sediments resuspended in PBS, and the ultracentrifugation step repeated. All centrifuge steps were performed at 4 °C.

### Sucrose gradient separation

For sucrose density centrifugation, the gradient was layered by pouring sequentially 2 ml of each of the 6 sucrose solutions (from 2.0 M to 0.25 M) in 0.35 M increments. The pellet was resuspended in 2 ml of 0.95 M sucrose solution and then placed into the sucrose step gradient column. Tubes was centrifuged at 200,000 × *g* for 16 h at 4 °C. A total of 7 fractions (1–7) were collected and resuspended in cold PBS, and centrifuged at 100,000 × *g* at 4 °C for 90 min. Seven sucrose gradient fraction pellets were resuspended in lysis buffer for further western blot analysis.

### Transmission electron microscope (TEM)

TEM (HT7700, Hitachi) was used to observe the ultrastructure of EVs. Briefly, an aliquot of 10 μl of the CEC-EVs was dropped on carbon‐coated copper grids and adsorbed for 1 min. After removing excess liquid with filter paper, the samples were subjected to negative staining for 1 min by using 2% uranyl acetate at room temperature. Once again, use filter paper to remove remaining liquid, and air-dry the stained sample until it is completely dry. Images were captured at 120 kV accelerating voltage.

### Nanoparticle tracking analysis (NTA)

Size distribution and concentrations of EVs were determined by using ZetaView PMX 120-Z (Particle Metrix, Meerbusch, Germany) and its corresponding software (ZetaView 8.5.16.1002). To calibrate the instrument before taking sample readings, a 1 µl volume of 100 nm standard polystyrene microparticles (Applied Microspheres) was diluted 250,000-fold. The instrument pre-acquisition parameters were configured as follows: temperature of 23 °C, sensitivity of 85, frame rate of 30 frames per second, shutter speed of 100 and laser pulse duration was matched to shutter duration. The post-acquisition parameters were adjusted to ensure a minimum brightness of 25, maximum size of 200 pixels, and minimum size of 10 pixels. For each sample, 1 μl of the resuspended EVs pellet was diluted into 1 ml of filtered 1× PBS, and manually loaded into the NTA sample chamber via a syringe. The particle count was measured at 11 different positions, with three cycles of reading at per position. After performing automated analysis and eliminating any outliers from the 11 positions, the machine software calculated the mean, median, and mode sizes, as well as the concentration of the sample.

### EVs tracking in vivo

To verify the EVs distribution in vivo, purified EVs were incubated with the lipophilic near-infrared red dye DiR for 30 min at 37 °C. Free DiR was removed by another round of EVs isolation. Then DiR-labeled EVs were injected into mice via the tail vein. Mice were sacrificed and the organs were harvested 24 h later. The in vivo imaging system (IVIS Spectrum, PerkinElmer, USA) was used to quantify DiR dye signals in each organ.

The purified CEC-EVs were labeled with PKH67 green fluorescence membrane dye (Sigma, Shanghai) according to the manufacturer’s instructions. Labeled CEC-EVs pellet were washed with PBS and harvested by ultracentrifugation, and resuspended in PBS again. For in vivo tracking EVs, the labeled EVs (20 ug) were injected via the tail vein in a total volume of 100 μl PBS. Brain tissue was collected for immunofluorescence analysis 24 h after injection.

### Transwell assay

To determine whether CEC-EVs can be efficiently taken up by activated microglial cell, a transwell co-culture system was established. The CECs were seed in the upper layer of a transwell chamber and cultured in DMEM medium containing Exosome-free FBS. The lower layer was 600 ul DMEM medium which contained 10% Exosome -free FBS inoculated with BV2 cells. The transwell plates was placed in 37 °C incubator with 5% CO_2_. After 24 h of cultivation, BV2 cells on the lower chamber were fixed with 4% paraformaldehyde and images were obtained with inverted microscope (Olympus IX 73). To investigated the effect of miRNA and CEC-EVs on inflammatory stimuli, BV2 cells were seeded into the upper chambers of Matrigel-coated filters. The bottom chambers were filled with 0.5 ml of DMEM medium as a chemoattractant. After incubation for 12 h at 37 °C, the cells in the upper surface of the chamber were cleaned carefully with cotton swab. Then cells invaded to the lower chamber were fixed with methanol, stained with 0.5% crystal violet, and counted under the microscope.

### Transfection

The miR-672-5p mimic, negative control (miR-672-5p -NC), cy3-lablled miR-672-5p and small interfering RNAs (siRNAs) targeting TAB2 were synthesized from GenePharma (Shanghai, China) and transfected into BV2 cells. Cells were seeded in plates 1 day before transfection to reach about 70% confluency at the day of transfection. The transfection procedure was performed using Lipofectamine RNAiMAX (Invitrogen) according to the manufacturer’s instructions.

### Dual-luciferase reporter assay

The 3′-UTR or mutated miR-672-5p binding site of TAB2 subcloned into the pmirGLO Dual-luciferase Target Expression Vector (Promega, Madison, WI, USA) to construct the reporter vector pmirGLO-TAB2-WT and pmirGLO-TAB2-Mut. miR-672-5p mimic or mimic-NC were transfected to BV2 cells, followed by transfection with pmirGLO-TAB2-MT or pmirGLO-TAB2-Mut plasmid. The luciferase activity was determined by luciferase activity kit (promega) according the manufacturer’s protocol.

### Co-immunoprecipitation

The Co-immunoprecipitation (Co-IP) assay was performed using Co-IP kit (Absin Bioscience, China) according to the manufacturer’s instructions. The BV2 cells was transfected with the Flag‐TAK1 and/or MYC-TAB2 plasmid. Then, cell lysates were incubated with anti‐MYC antibody (ab32, Abcam) overnight at 4 °C. Samples were immunoprecipitated with anti‐MYC antibody overnight at 4 °C. Proteins were eluted from the beads and separated by SDS-PAGE. For without tag Co-IP assay, the cell and tissue lysates were incubated with anti‐TAB2 antibody (ab264309, Abcam) overnight at 4 °C. Samples were immunoprecipitated with anti‐TAB2 antibody overnight at 4 °C. Proteins were eluted from the beads and separated by SDS-PAGE.

### Proximity ligation assay

PLA experiment was conducted using Duolink PLA kit (Sigma) according to the manufacturer’s instructions. Briefly, the cells were cytospun onto glass slides and fixed with 4% PFA. Following permeabilization with 0.2% Triton X-100 for 5 min, the cells were blocked with Duolink blocking buffer for 30 min. Subsequently, cells were incubated with primary antibodies (anti-TAB2 and anti-TAK1) for overnight at 4 °C. The PLA probes were diluted with Duolink® antibody diluent in appropriate proportions, followed by ligation and amplification. The slides were washed with buffer and then mounted using Duolink in situ mounting medium containing DAPI. The slides were observed under a fluorescence microscope (Olympus). For tissue, sections were dewaxed in xylene, hydrated through gradient ethanol and antigen retrieval was performed. Following antigen retrieval, the sections were incubated with primary antibodies for overnight at 4 °C. The remainder of the procedure was the same as that used for the cells.

### Enzyme-linked immunosorbent assay and NFκB activity analysis

TNF-α ELISA kit (MTA00B), IL-1β ELISA kit (MLB00C), IL-6 ELISA kit (M6000B,) and IL-10 ELISA kit (M1000B) were purchased from R&D Systems. All operations were performed in accordance with the manufacturer’s protocols provided by the kit. NFκB activity was detected using Quanti-blue assay following the manufacturer’s protocols (InvivoGen).

### Real-time quantitative PCR analysis

Trizol reagent (Invitrogen, Shanghai, China) was used to extract total RNA from tissues and cells according to the manufacturer’s instructions. The final RNA concentration was measured using the NanoDrop 2000 spectrophotometer (Thermo Scientific). Total RNA was reverse transcribed into cDNA using SuperScript III Reverse Transcriptase (Invitrogen, Shanghai, China). Quantitative real-time PCR was performed on a CFX96 Touch Real-Time PCR detection system (Bio-Rad). The expression level of β-actin was used as the internal standard, and the experiments were repeated three times with triple samples. The primers were ordered from Origene and sequences of the primers used are listed in Table S1.

### Western blot

Total protein of tissues and cells was extracted using the M-PER mammalian protein extraction reagent (78501, Thermo Scientific). The EVs protein was extracted using a Total Exosome RNA and Protein Isolation Kit (4478545, Invitrogen). NE-PER Nuclear and Cytoplasmic Extraction Reagents (78835, Thermo Scientific) was used for extraction of nuclear protein and cytoplasmic protein. Protein concentrations were determined following the instructions of Pierce BCA Protein Assay Kit (23227, Pierce). Protein was separated by 10% SDS-PAGE and transferred to polyvinylidene difluoride membranes (PVDF). The PVDF membrane was then blocked with 5% nonfat milk at room temperature for 2 h and then incubated overnight at 4 °C with primary antibodies against syntenin (ab19903, Abcam), Alix (ab186429, Abcam), CD63 (ab217345, Abcam), TSG101 (ab125011, Abcam), GRP94 (GP96, ab32568, Abcam), Calnexin (ab22595, Abcam), TAB2 (DF2368, Affinity), TAK1 (AF7616, Affinity), p-TAK1 (AF3019, Affinity), IKKβ (ab32135, Abcam), p-IKKβ (AF3010, Affinity), IκBα (AF5002, Affinity), p-IκBα (AF2002, Affinity), NFκB (AF5006, Affinity), LC3 (ab192890, Abcam), Beclin 1 (ab62557, Abcam), SQSTM1/p62 (ab109012, Abcam), β-actin (ab8227, Abcam), PCNA (ab18197, Abcam). The secondary antibodies used were horseradish peroxidase (HRP)-conjugated goat anti-rabbit IgG (ab6721, Abcam). Gene expression levels were normalized to those of β-actin or PCNA and the signals were quantified using ImageJ software.

### Immunofluorescence

Immunofluorescence staining were performed using Tyramide Signal Amplification (TSA) technology. Paraffin-embedded cortical tissues were cut into 4-μm thick sections. The sections were dewaxed in xylol and rehydrated in graded ethanol series. Then, sections were immersed in EDTA antigen retrieval buffer for antigen retrieval. To block endogenous peroxidase, sections were exposed to 3% H_2_O_2_ for 20 min. For immunocytochemistry, cultured cells were fixed on 4% formaldehyde, permeabilized with 0.5% Triton X-100 for 20 min. Cells were blocked using 3% BSA at room temperature. Then, the sections or fixed cells were incubated with first primary antibodies for overnight at 4 °C and incubated with HRP-conjugated secondary antibody for 30 min at room temperature followed by Cy3-TSA solution treatment. Sections or fixed cells were submerged in EDTA buffer (pH 8.0) and microwaved to remove the primary antibody and secondary antibody. Then sections or fixed cells were again incubated with second primary antibody, secondary antibody and FITC-TSA solution. Nuclei were counterstained with 4,6-diamidino-2-phenylindole (DAPI). Antibodies used were as follows: IBA-1 (ab178847, Abcam), CD68 (ab53444, Abcam), NFκB (AF5006, Affinity), LC3 (ab192890, Abcam), Beclin 1 (ab62557, Abcam), NLRP3 (DF7438, Affinity), iNOS (ab178945, Abcam), ARG-1 (DF6657, Affinity), HRP-conjugated goat anti-rabbit IgG (ab6721, Abcam).

### Behavioral tests

Forced swim test: the FST was performed as previously described [52]. C57BL/6J mice were placed in a transparent plexiglass cylinder (height: 30 cm, diameter: 10 cm) filled with 15 cm of water. The mice were forced to swim for 6 min and the immobility time was recorded in the last 5 min. If the mouse was floating in the water without struggling, and makes only those movements necessary to keep its head above the water surface, the mouse was considered immobile.

Sucrose preference test (SPT): before the test, two bottles of 1% sucrose solution were placed on each side of the cage, and mice were trained to habituated to sucrose solution for 24 h. Then one bottle of sucrose solution was replaced with water for another 24 h. After the adaptation stage, mice were deprived of food for 24. Each animal was free access to two pre-weighed bottles simultaneously, one containing 1% sucrose solution and the other containing equal weight tap water. In order to avoid spatial bias, The position of two bottles were randomly placed on the left and right side. After 12 h, the weight of consumed sucrose solution and water were recorded. The sucrose preference was calculated with the following formula: sucrose preference (%) = (sucrose solution consumption/total liquid consumption) × 100%.

Elevated plus maze test: the EPM was performed as previously described [53]. The EPM apparatus consisted of two opposing open arms (35 × 10 cm) which were perpendicular to two opposing closed arms (35 × 10 cm) with a small central square (5 × 5 cm) between the arms. Two closed arms were delimited by vertical walls, while the open arms had unprotected edges. Mouse was gently placed at the center of the platform, facing toward an open arm, and allowed to freely explore for 5 min. The total number of entries into the open arm and closed arm as well as the time spent in the open arm during the test were recorded via a video camera.

Morris water maze test (MWM): the MWM experiment was used to evaluated the spatial memory and learning ability and the experiment lasted for 6 days in total. The MWM was a round tank that divided into four equally sized imaginary quadrants, with a round platform submerged 1 cm beneath surface of the water at the center of one quadrant. The navigation test was performed in the first 5 days. For each trial, mouse was put in the tank facing the wall from four different point, and allowed 60 s to find the escape platform. Once the mice reached to the platform, the time was record as escape latency. If the mice were unable to find the platform within the given time, it was guided to platform and stay for further 10 s, the escape latency was recorded 60 s. After 24 h, the platform was removed and mice were released from the opposite quadrant for spatial probe test. The time spent in the target quadrant, and swim speed of mice were recorded. Investigators were blinded to individual/group during data analysis.

### Nissl staining, hematoxylin and eosin staining and Tunel assay

Nissl staining was performed according to routine protocols. After dewaxing, sections were immersed in 1% Cresyl Violet for 10 min at room temperature. The sections were washed with distilled water and differentiated with 95% ethanol. Finally, the sections were permeabilized with xylene, sealed with neutral resin and observed under inverted optical microscope. For HE staining, brain tissue sections were deparaffinized in xylol 20 min for twice, and then rehydrated in absolute ethanol 5 min for twice, 75% ethanol 5 min and tap water for 5 min. Tissue sections were stained with hematoxylin and eosin. After dehydration with graded alcohol and clearing in xylene, the sections were then observed by using Olympus IX73 microscope. TUNEL staining were performed using TUNEL assay kit (Beyotime, China). Paraffin-embedded brain sections were deparaffinized, treated with 20 µg/ml proteinase K without DNase at 37 °C for 20 min. After three times PBS washes, sections were then stained with 50 μl TUNEL reaction mixture and incubated for 60 min at 37 °C, protected from light. Sections were sealed with anti-fluorescence quenching sealing tablets and imaged using fluorescence microscope (IX73, Olympus).

### Statistical analysis

Data were analyzed using SPSS v.13.0 software (SPSS Inc., Chicago, IL, USA) and the results are presented as means ± SD. Statistical analysis includes unpaired Student’s *t* test and one-way ANOVA. Data were performed using GraphPad Prism 7 software (GraphPad Software). *p* values < 0.05 was significant.

### Supplementary information

Supplementary materials

Original Data File

## Data Availability

The data that support the findings of this study are available from the corresponding author upon reasonable request.
