# Supplementary material for: Cerebral endothelial cell-derived extracellular vesicles regulate microglial polarization and promote autophagy via delivery of miR-672-5p
Source: Cell Death Dis. 2023 Sep 29;14(9):643. doi: 10.1038/s41419-023-06173-5 (PMC10541416; doi:10.1038/s41419-023-06173-5)
Supplement: Supplementary file 1 — Supplementary materials [file 41419_2023_6173_MOESM1_ESM.docx]

**Supplementary materials**

**Cerebral endothelial cell-derived extracellular vesicles** **regulate microglial polarization and promote autophagy via delivery of miR-672-5p**

**This file includes:**

- Supplementary Fig. 1-7
- Supplementary Table 1

**Supplementary Figures**


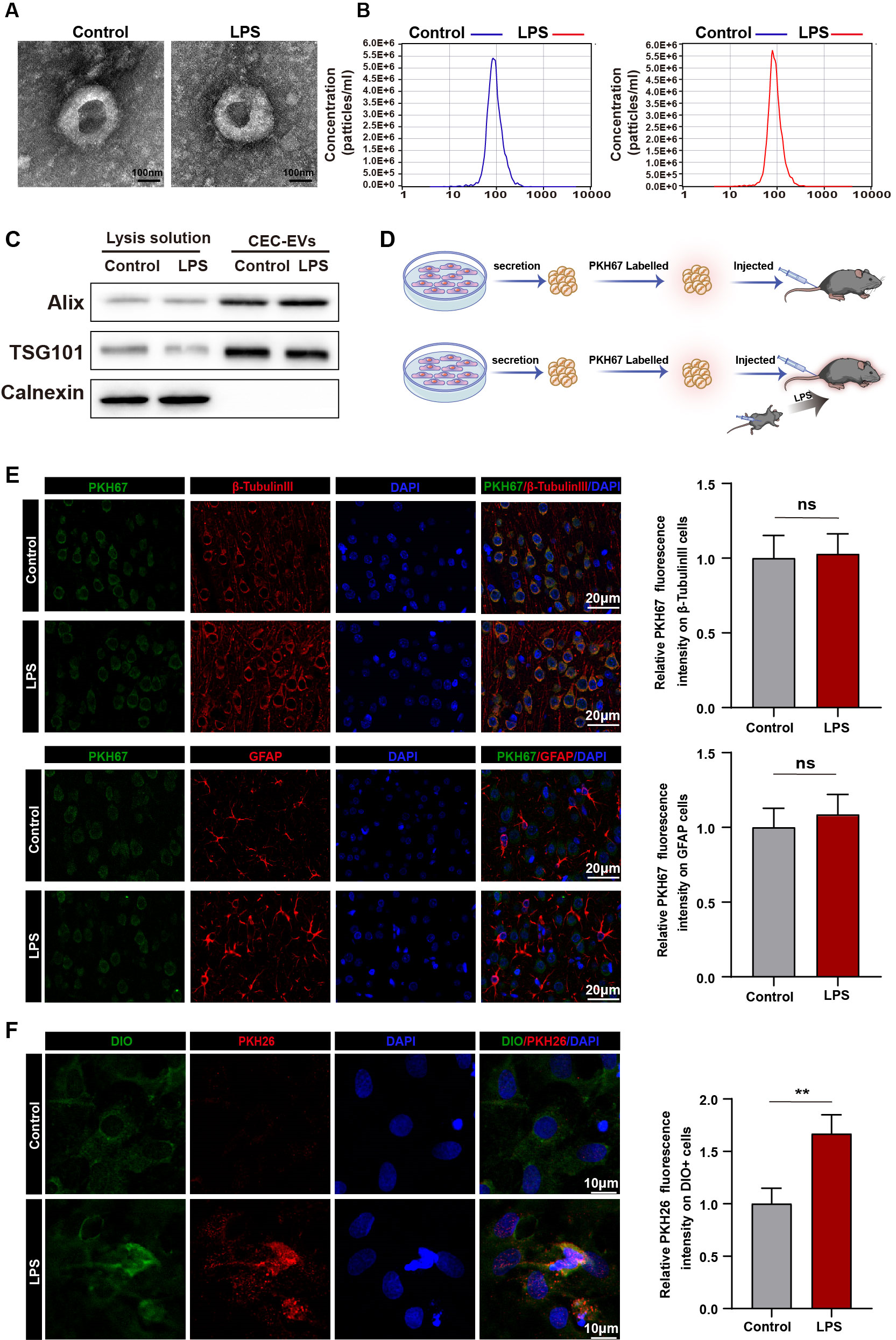


**Fig. S1.** CEC-EVs could be successfully absorbed by neural cells and the absorptive process was changed by neuroinflammatory activation. (**A**) The shape of CEC-EVs were observed by TEM. (**B**) The concentration and size of CEC-EVs were analyzed by NTA. (**C**) EVs markers Alix and Tsg101 and negative protein Calnexin were performed using Western blot. (**D**) The schematic diagram of the *in vivo* assay used to verify whether CEC-EVs can be absorbed by neural cells. (**E**) Immunofluorescence co-stain of PKH-67 with βIII-tubulin and GFAP. (**F**) LPS treatment increased microglial uptake of CEC-EVs. The lipid membrane of CECs was stained with PKH26 red fluorescent dye and the cells were co-cultured with primary microglial cells stained with DIO in a Transwell. All data are present as means ± SD (n = 3). Unpaired Student’s t-test. ns=not significant compared to Control group. ^**^P < 0.01 compared to Control group.


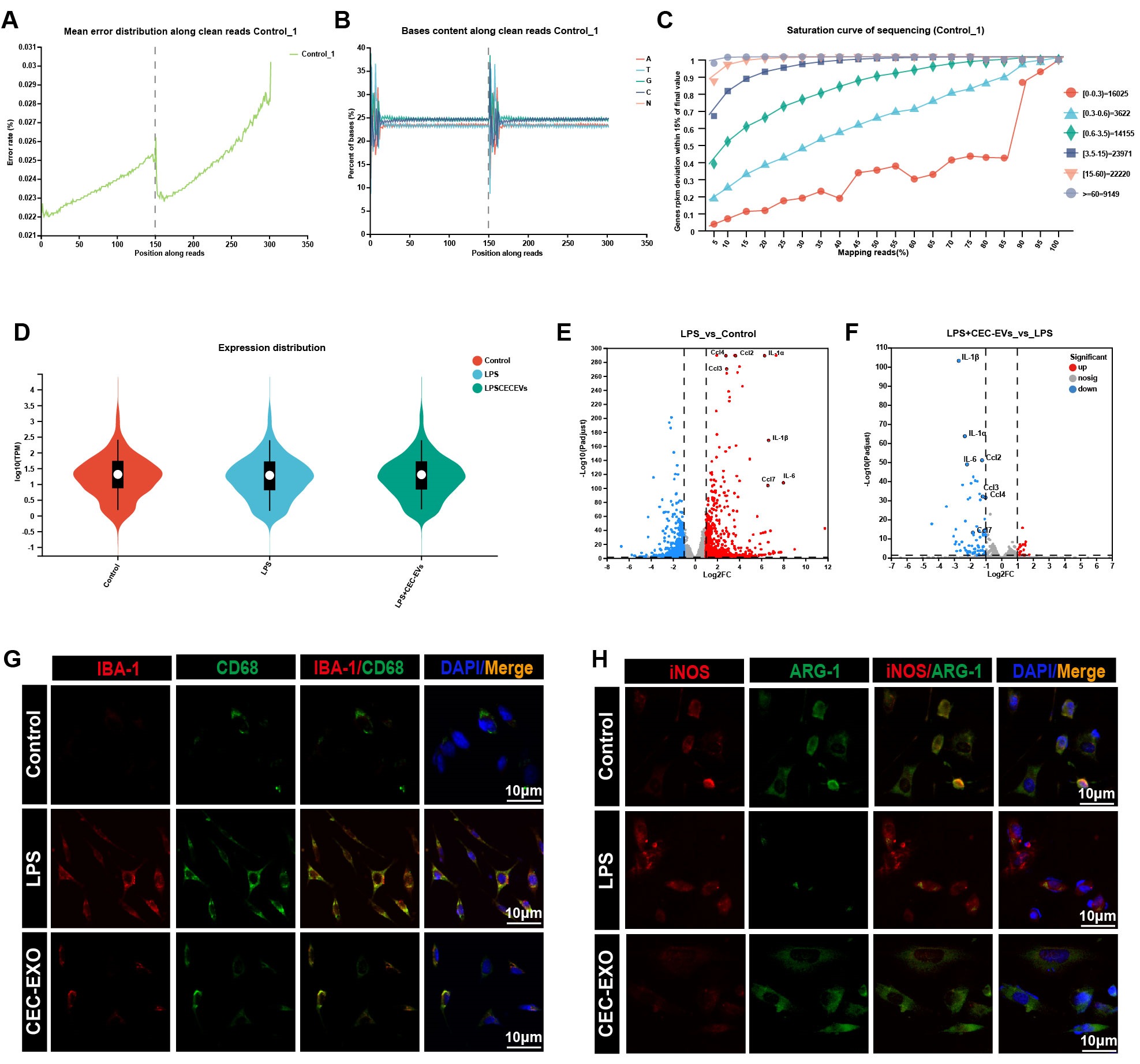


**Fig. S2.** CEC-EVs alleviated LPS-induced inflammatory response and shifted the microglial polarization. **(A-D)** Error distribution (**A**), bases content (**B**), gene expression distribution (**C**), and saturation curve (**D**) of the transcriptomic data. **(E and F)** Volcano map and heat map of differentially expressed genes. (**G**) CEC-EVs alleviates LPS-induced microglial activation. (**H**) Immunofluorescence of iNOS and ARG-1.


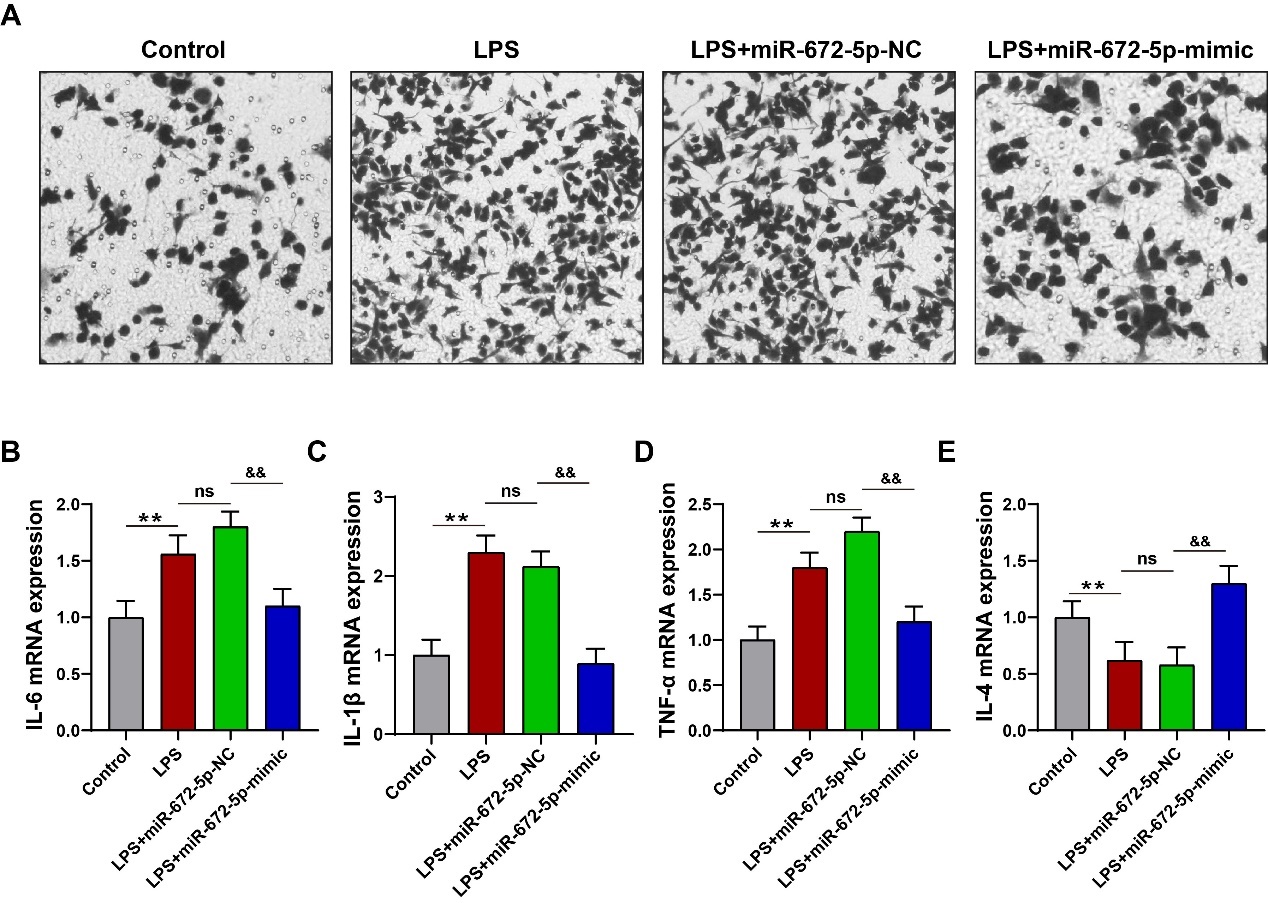


**Fig. S3.** miR-672-5p promoted cell migration and alleviated the LPS-induced inflammation. (**A**) miR-672-5p mimic inhibited migration cell migration. (**B-E**) miR-672-5p mimic alleviate the of LPS-induced inflammation. All data are present as means ± SD (n = 3). one-way ANOVA. ^**^P < 0.01 compared to Control group. ns=not significant compared to Control group. ^&&^P < 0.01 compared to LPS+miR-672-5p-NC group.


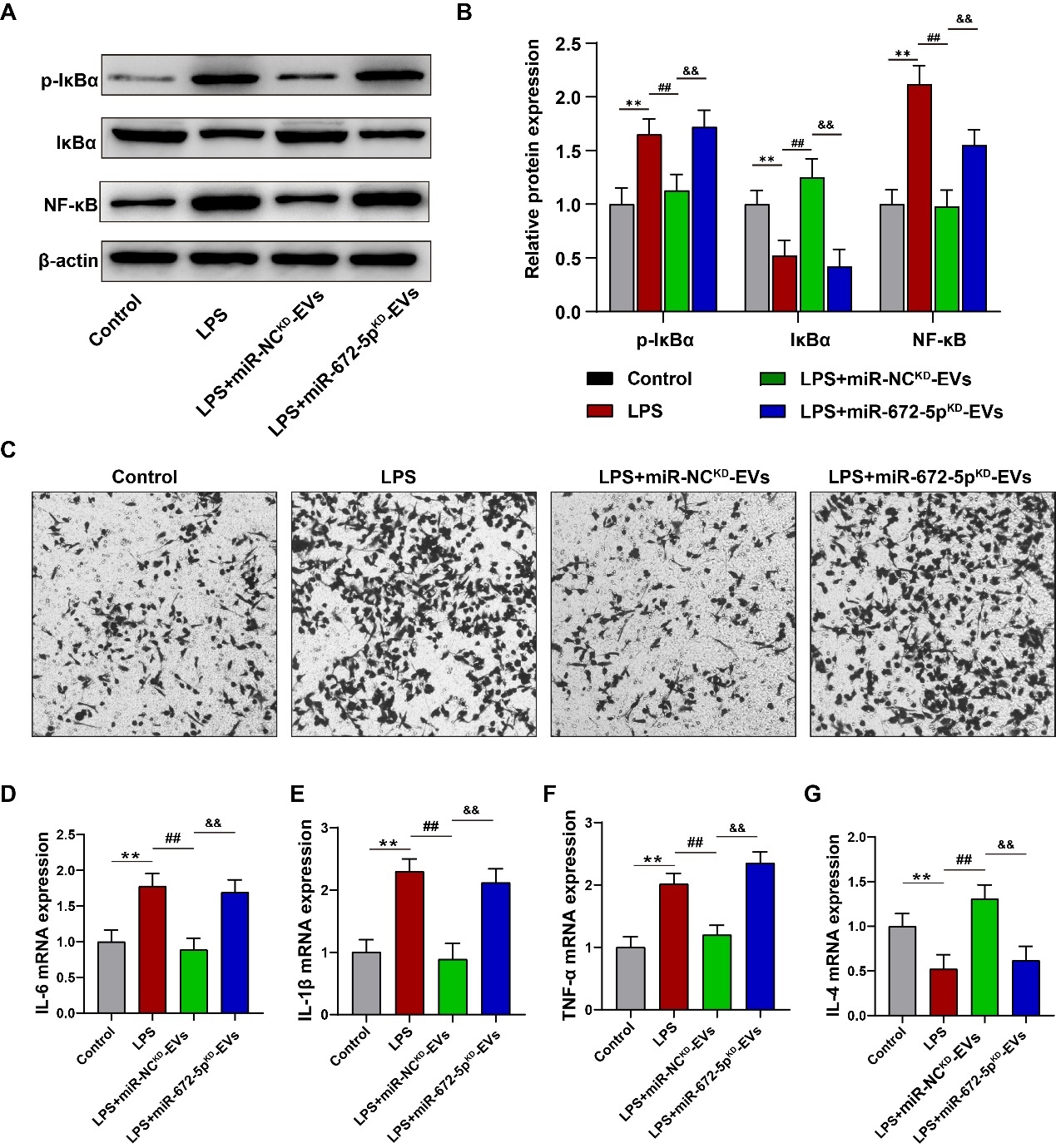


**Fig. S4.** CEC-EVs-derived miR-672-5p suppressed NFκB activation and alleviated LPS-induced inflammation *in vitro*. (**A**) Representative western blots of p-IκBα, IκBα, NFκB. (**B**) Statistical graphs of protein expression of p-IκBα, IκBα, NFκB. (**C**) CEC-EVs -derived miR-672-5p inhibited migration cell migration. (**D-G**) CEC-EVs-derived miR-672-5p alleviate LPS-induced inflammation. All data are present as means ± SD (n = 3). one-way ANOVA. ^**^P < 0.01 compared to Control group. ^##^P < 0.01 compared to LPS group. ^&&^P < 0.01 compared to LPS+miR-NC^KD^-EVs group.


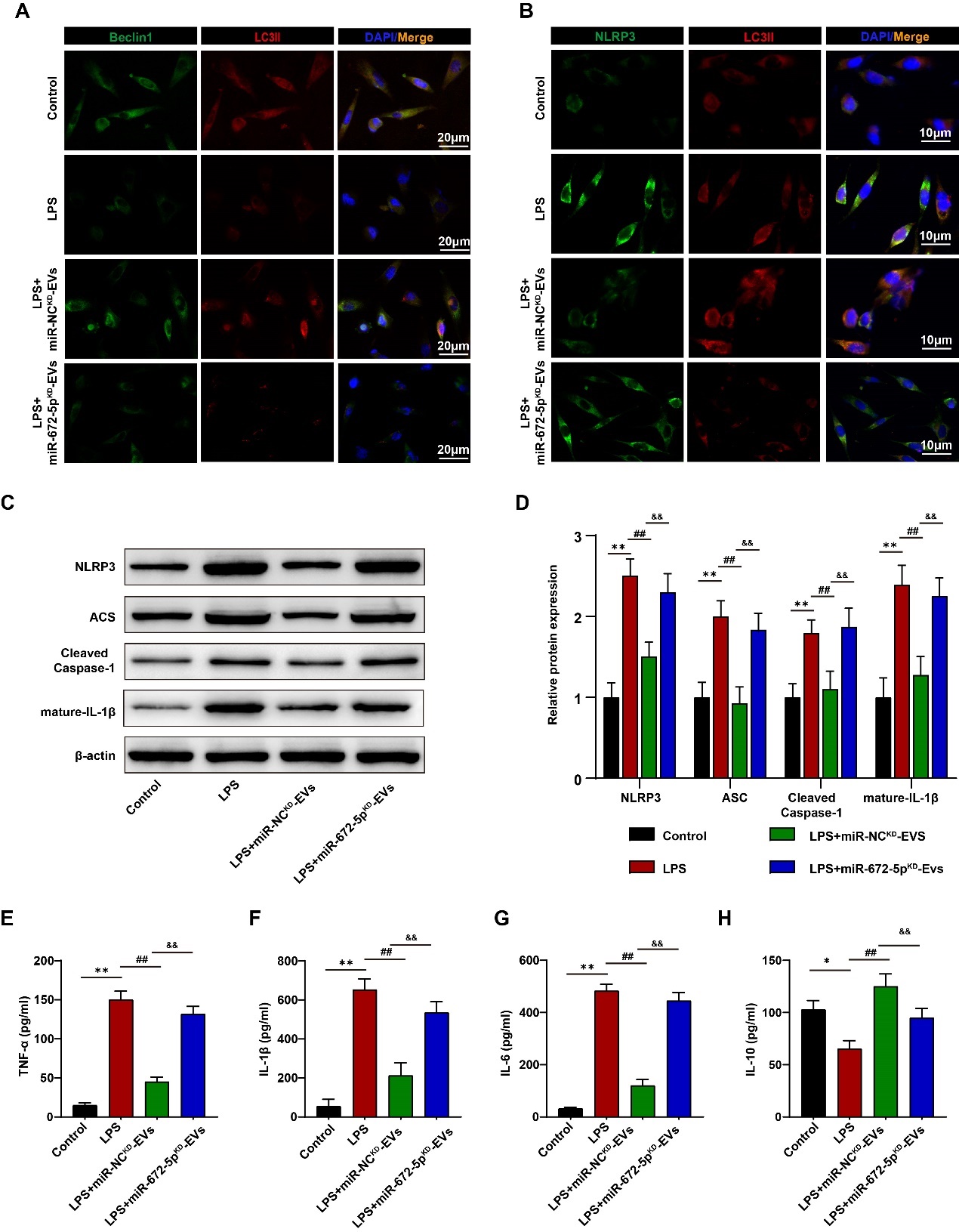


**Fig. S5.** CEC-EVs-derived miR-672-5p inhibited inflammasome activation in microglia. (**A**) Immunofluorescence staining of Beclin1 and LC3II. (**B**) Immunofluorescence staining of NLRP3 and LC3II. (**C**) Representative western blots of NLPR3, ASC, Cleaved-Caspase-1 and mature-IL-1β. (**D**) Statistical graphs of protein expression of NLPR3, ASC, Cleaved-Caspase-1 and mature-IL-1β. (**E-H**) CEC-EVs-derived miR-672-5p alleviate LPS-induced inflammation. All data are present as means ± SD (n = 3). one-way ANOVA. ns=not significant, ^**^P < 0.01 compared to Control group. ns=not significant, ^##^P < 0.01 compared to LPS group. ns=not significant, ^&&^P < 0.01 compared to LPS+miR-NC^KD^-EVs group.

**
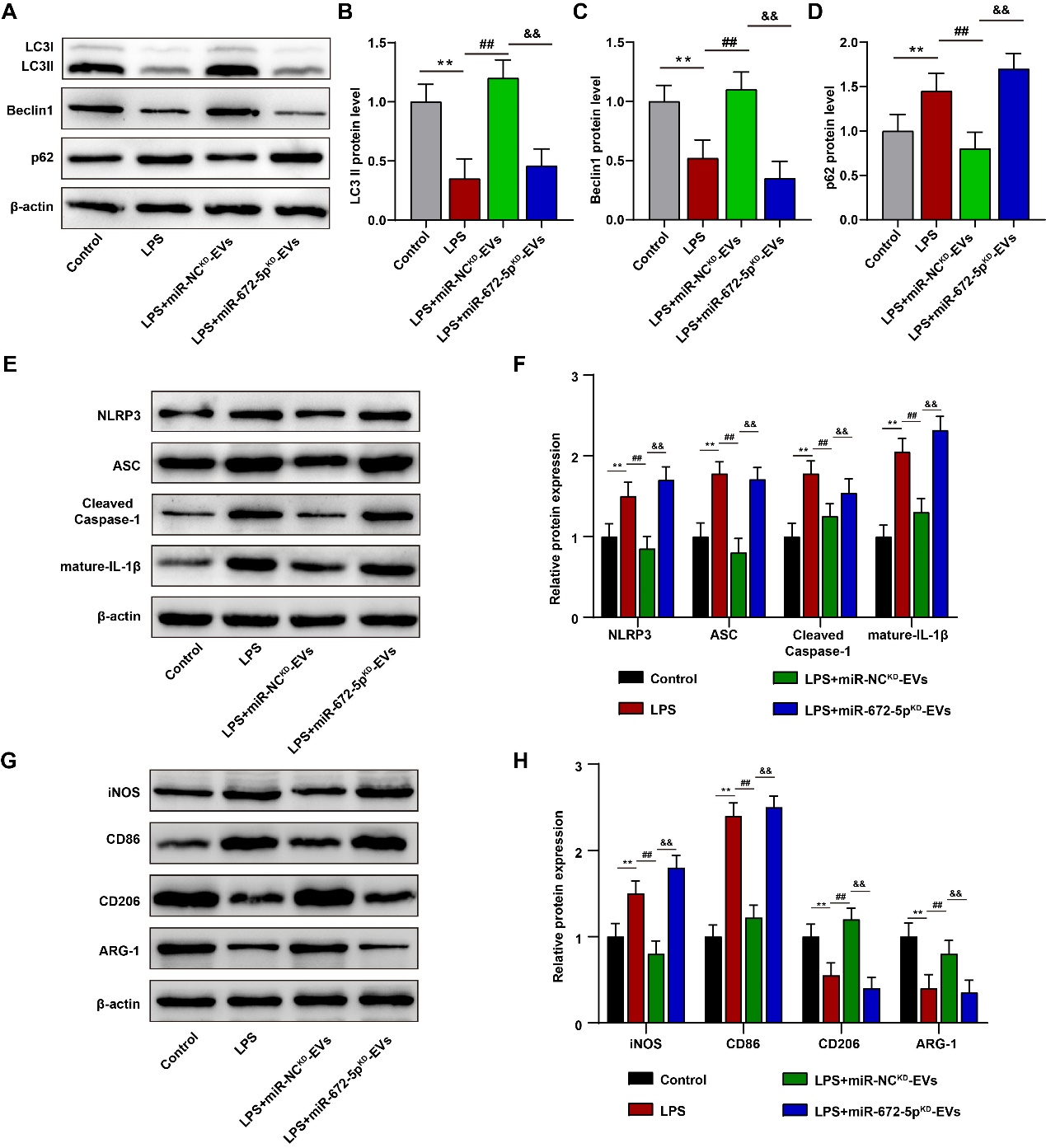
**

**Fig. S6.** CEC-EVs-derived miR-672-5p inhibited inflammasome activation *in vivo*. (**A**)

CEC-EVs-derived miR-672-5p enhances LC3II, Beclin1 level and decreases SQSTM1/p62 expression *in vivo*. (**B-D**) Statistical graphs of protein expression of LC3II, Beclin1 and SQSTM1/p62.) (**E**) Representative western blots of NLPR3, ASC, Cleaved-Caspase-1 and mature-IL-1β. (**F**) Statistical graphs of protein expression of NLPR3, ASC, Cleaved-Caspase-1 and mature-IL-1β. (**G**) Representative western blots of M1 marker (iNOS and CD86) and M2 marker (CD206, ARG-1). (**H**) Statistical graphs of protein expression of M1 marker and M2 marker. All data are present as means ± SD (n = 6). one-way ANOVA. ns=not significant, ^**^P < 0.01 compared to Control group. ns=not significant, ^##^P < 0.01 compared to LPS group. ns=not significant, ^&&^P < 0.01 compared to LPS+miR-NC^KD^-EVs group.

**
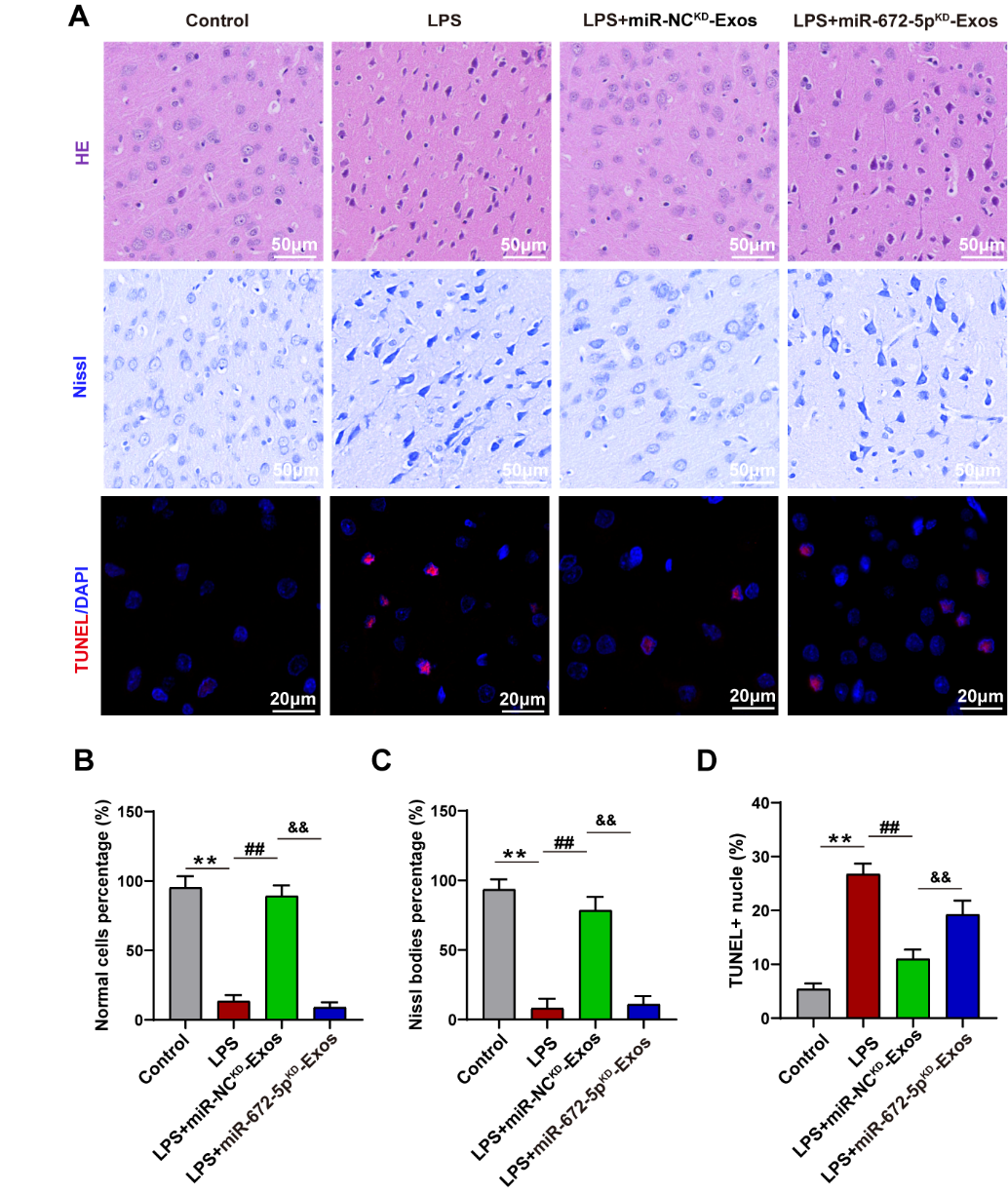
**

**Fig. S7.** CEC-EVs-derived miR-672-5p attenuated LPS-induced brain damage. (**A**) HE staining, Nissl staining and TUNEL test. (**B**) Quantitative of normal cells. (**C**) The percentage of Nissl bodies. (**D**) The number of TUNEL positive cells. All data are present as means ± SD (n = 6). one-way ANOVA. ^**^P < 0.01 compared to Control group. ^##^P < 0.01 compared to LPS group. ^&&^P < 0.01 compared to LPS+miR-NC^KD^-EVs group.

**Supplementary Table**

**Table S1:** Primer sequences used for qPCR analysis

| Gene | Sequences |  |
| --- | --- | --- |
| TNF-α | 5'- GGTGCCTATGTCTCAGCCTCTT -3' | Forward |
| TNF-α | 5'- GCCATAGAACTGATGAGAGGGAG -3' | Reverse |
| IL-1β | 5'- GTGTCTTTCCCGTGGACCTTC -3' | Forward |
| IL-1β | 5'- TCATCT CGGAGCCTGTAGTGC -3' | Reverse |
| IL-6 | 5'- TACCACTTCACAAGTCGGAGGC -3' | Forward |
| IL-6 | 5'- CTGCAAGTGCATCATCGTTGTTC -3' | Reverse |
| IL-4 | 5'- ATCATCGGCATTTTGAACGAGGTC-3' | Forward |
| IL-4 | 5'- ACCTTGGAAGCCCTACAGACGA-3' | Reverse |
| IL-10 | 5'- CGGGAAGACAATAACTGCACCC -3' | Forward |
| IL-10 | 5'- CGGTTAGCAGTATGTTGTCCAGC -3' | Reverse |
| β-actin | 5'-tactgctctggctcctagca-3' | Forward |
| β-actin | 5'- cggactcatcgtactcctgc-3' | Reverse |
