# Supplementary material for: Cerebral endothelial cell-derived extracellular vesicles regulate microglial polarization and promote autophagy via delivery of miR-672-5p
Source: Cell Death Dis. 2023 Sep 29;14(9):643. doi: 10.1038/s41419-023-06173-5 (PMC10541416; doi:10.1038/s41419-023-06173-5)

Figure 1D

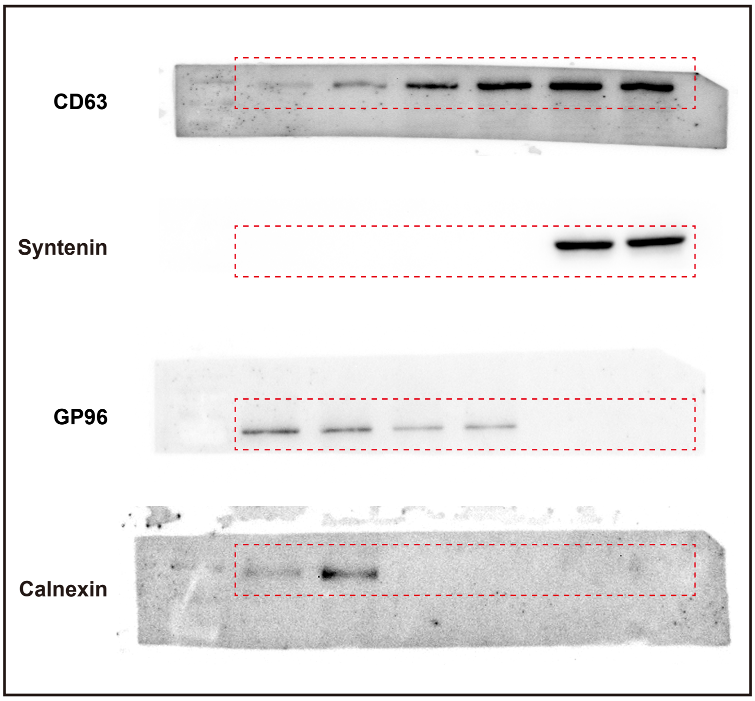

Figure 1F

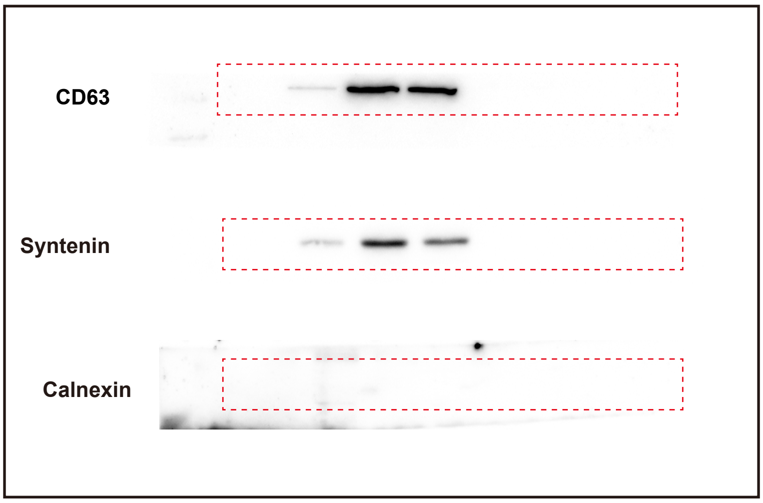

Figure 2G

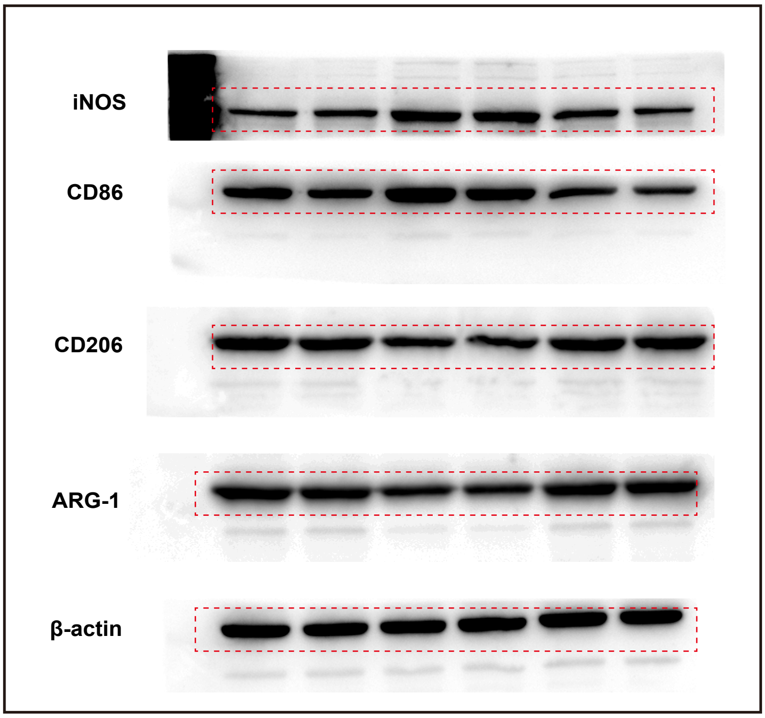

Figure 4E

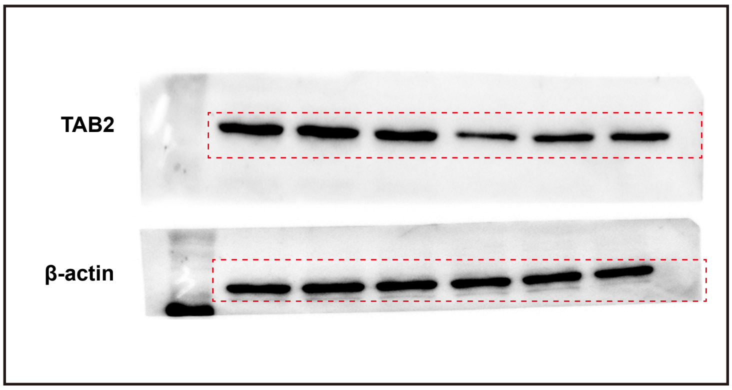

Figure 4H

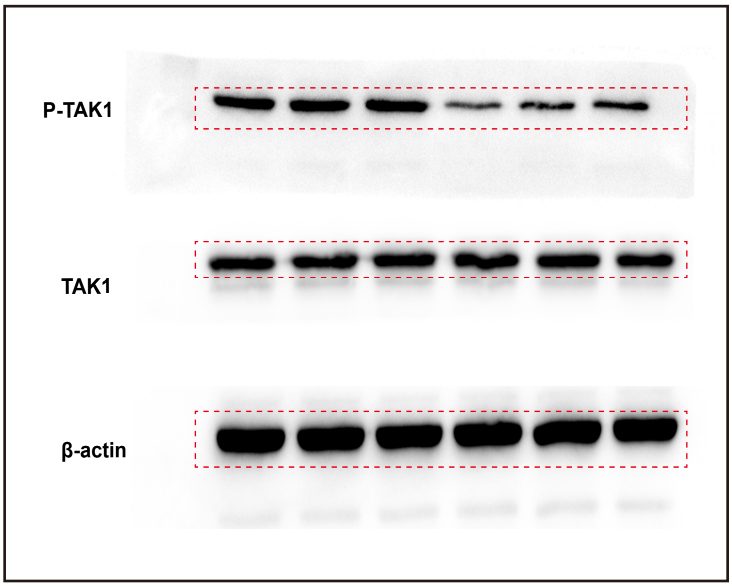

Figure 4I

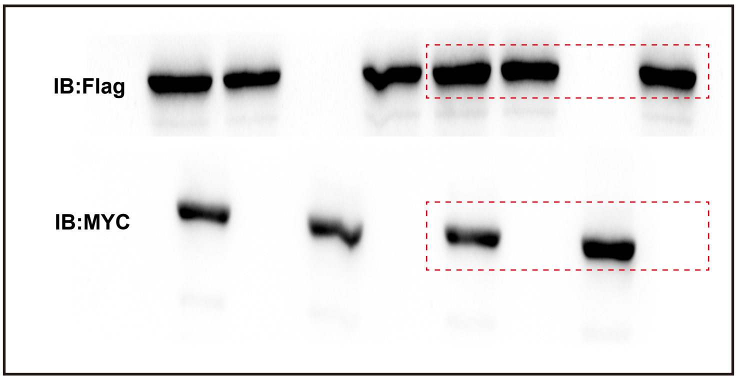

Figure 4J

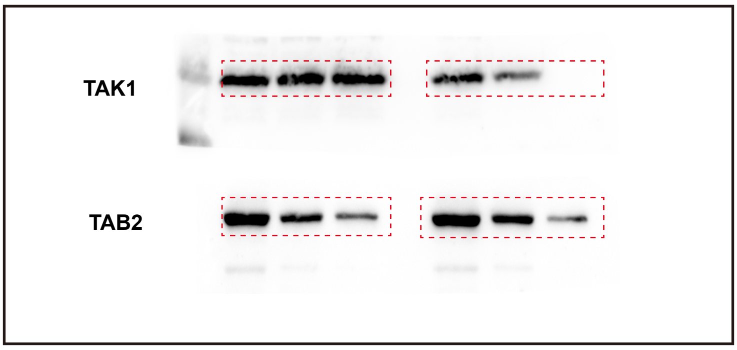

Figure 5A

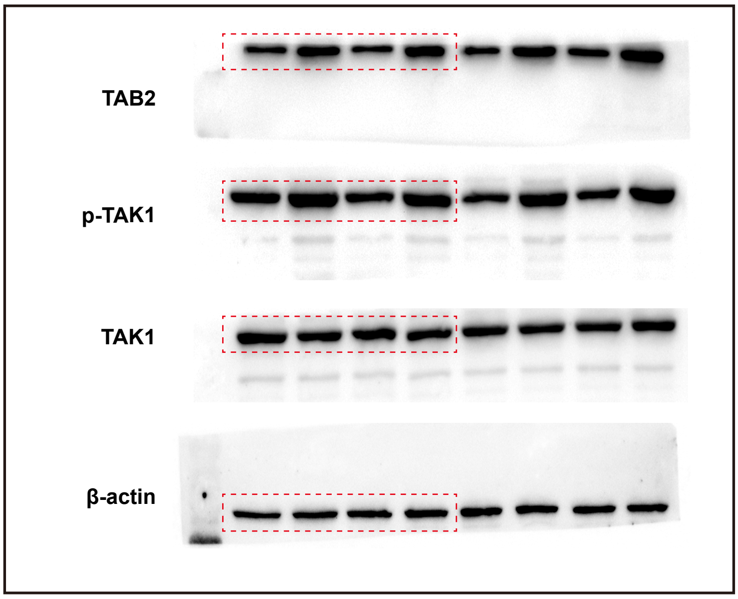

Figure 5B

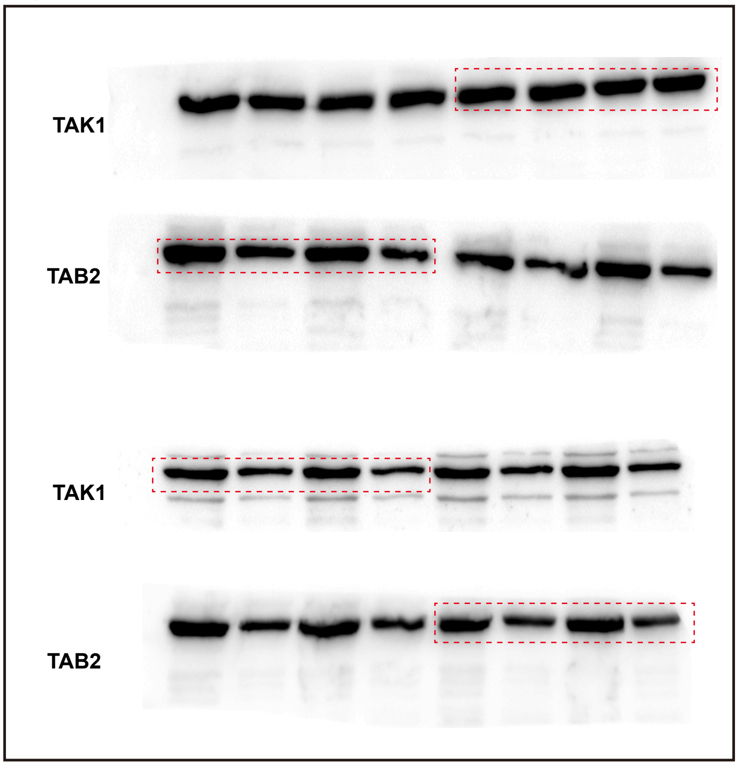

Figure 5E

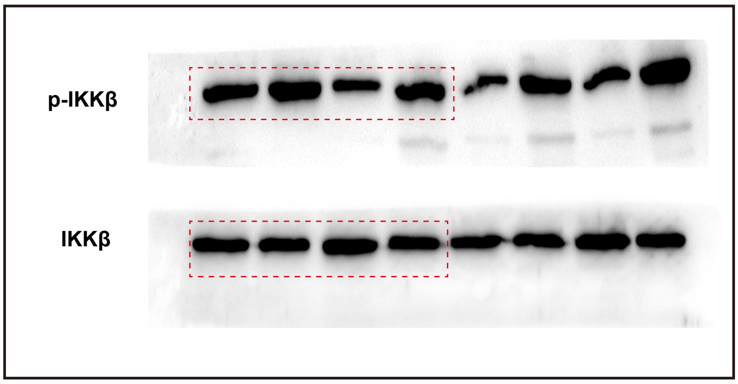

Figure 5F

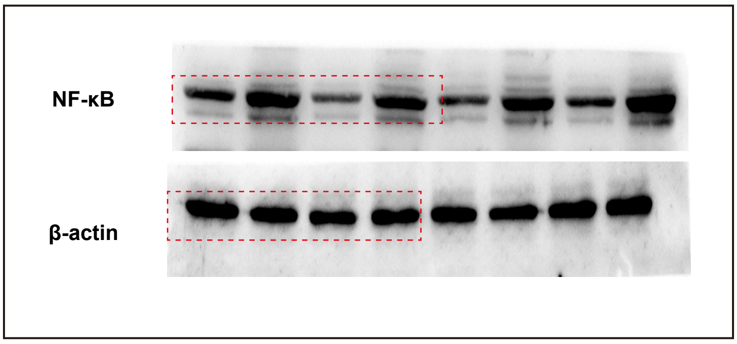

Figure 5G

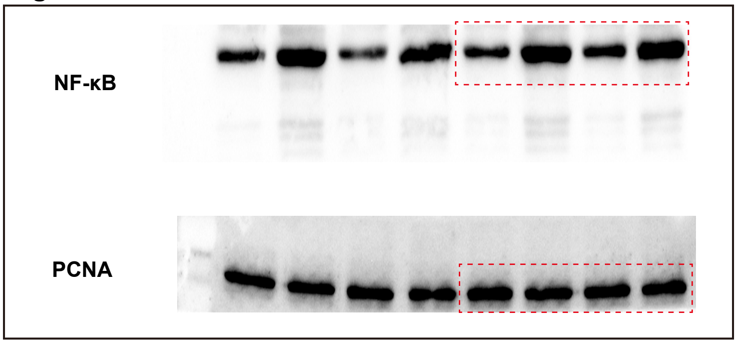

Figure 6B

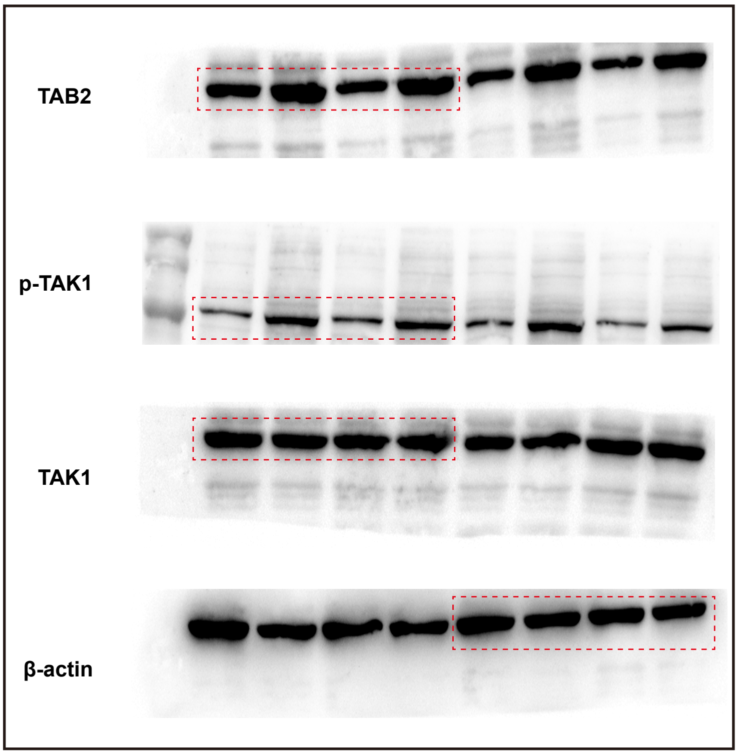

Figure 6D

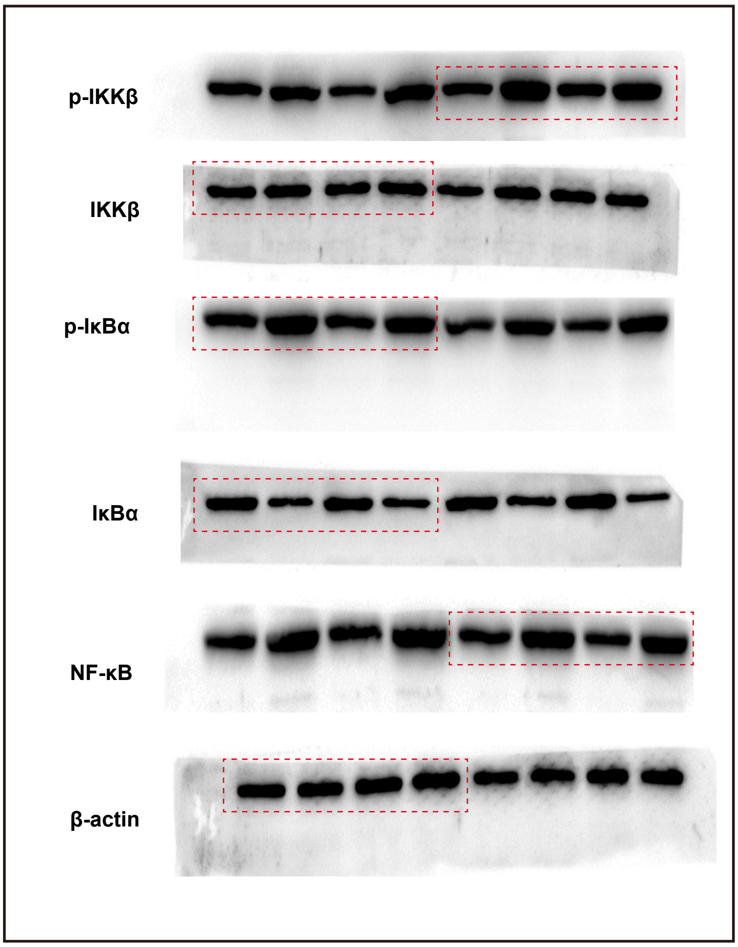

Figure 6H

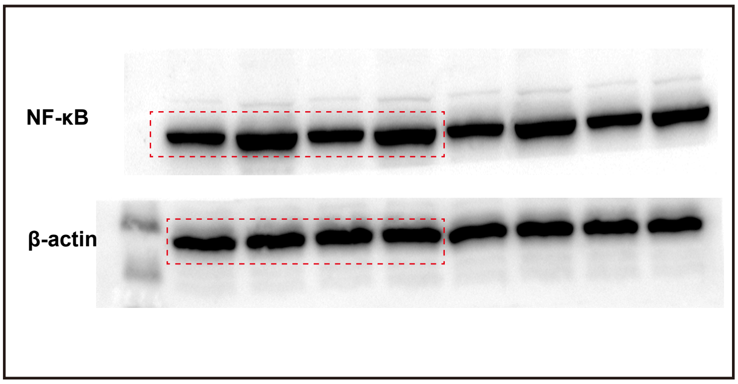

Figure 6I

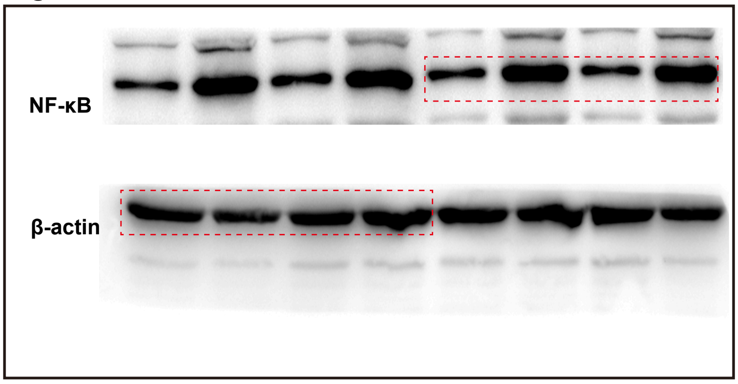

Figure 7A

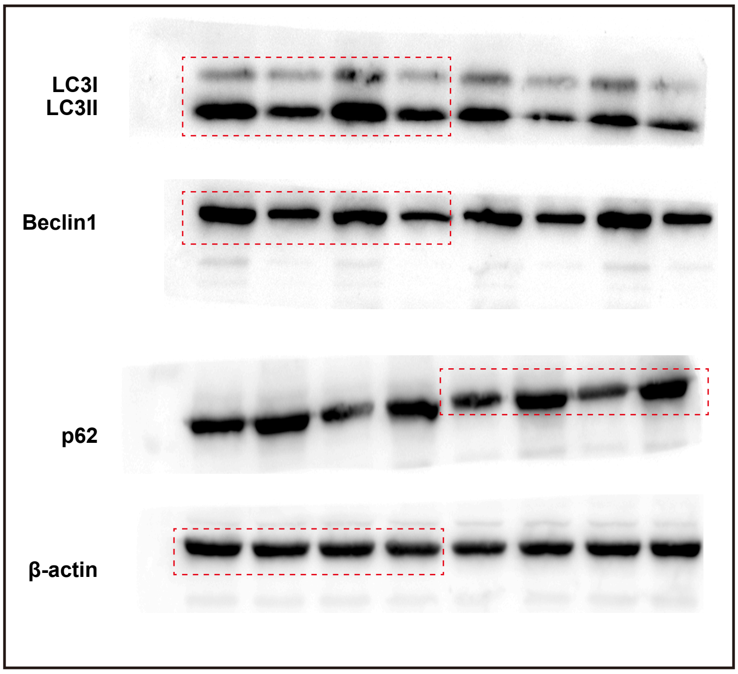

Figure 7H

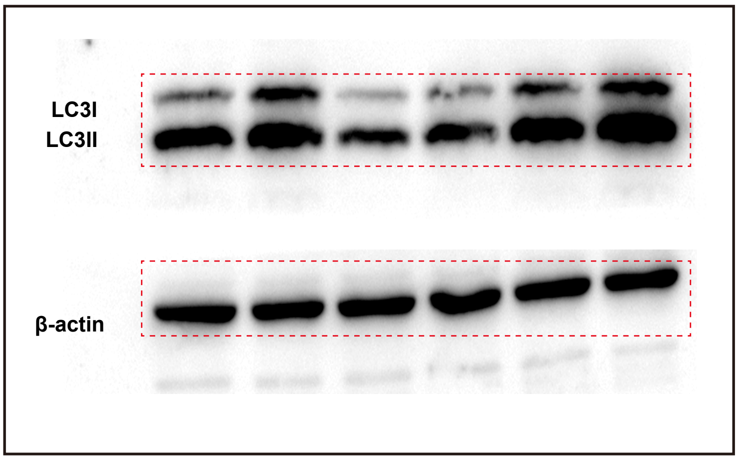

Figure S1C

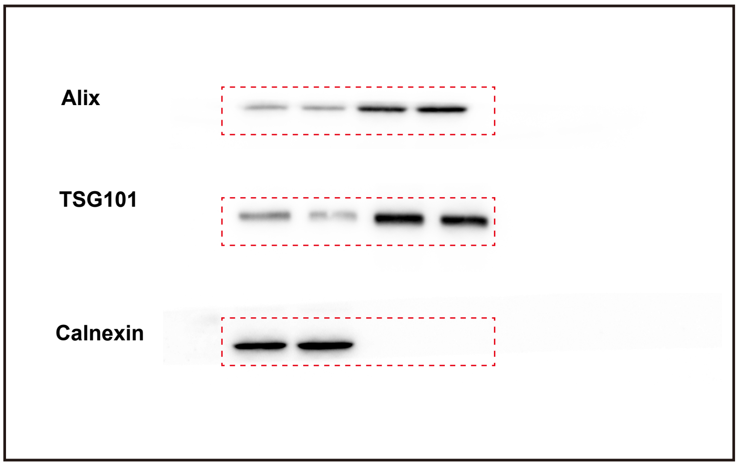

Figure S4A

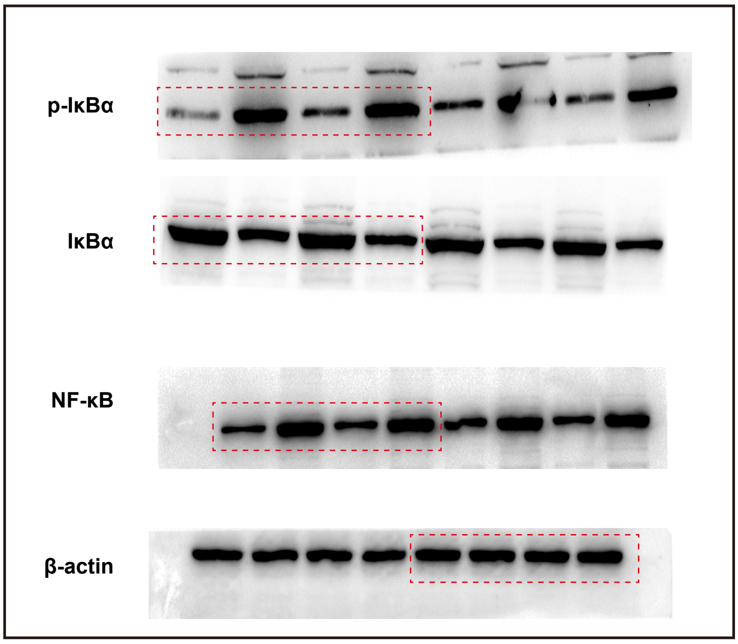

Figure S5C

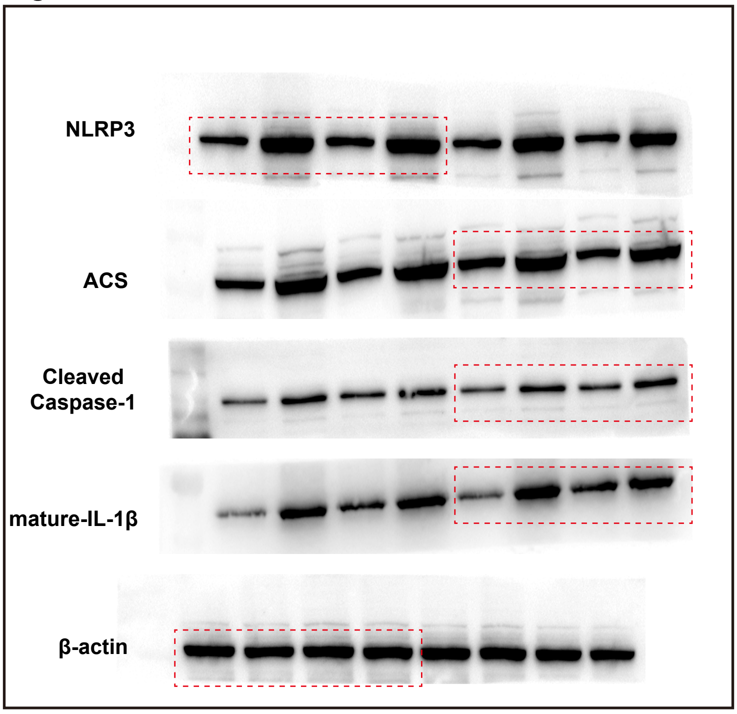

Figure S6A

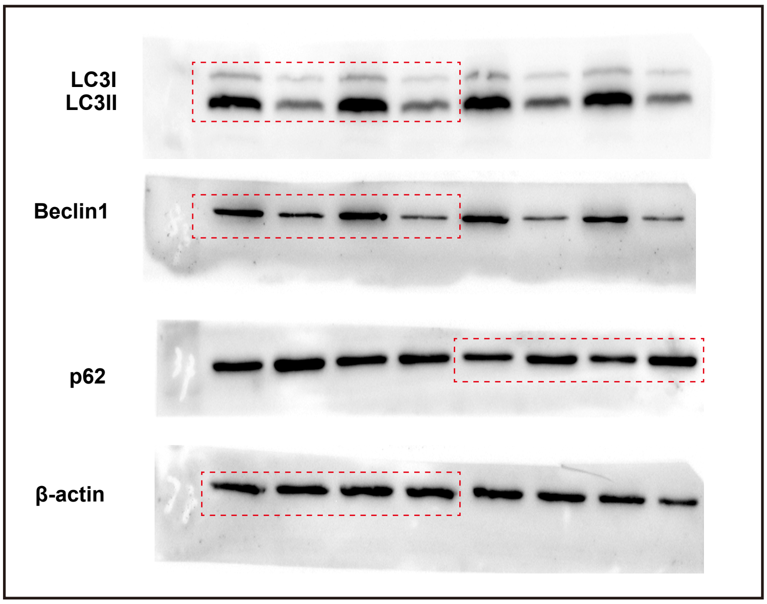

Figure S6E

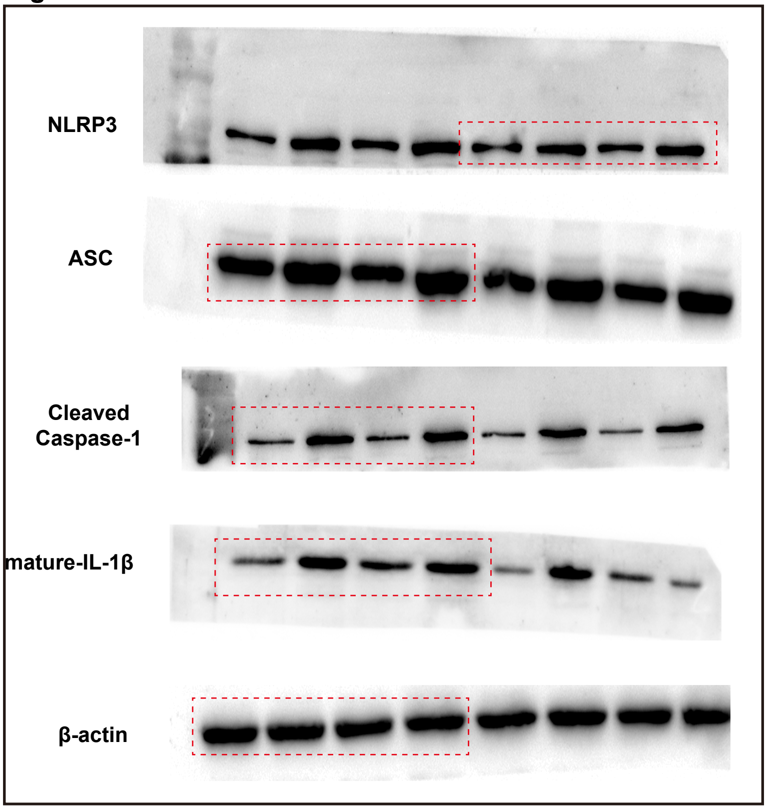

Figure S6G

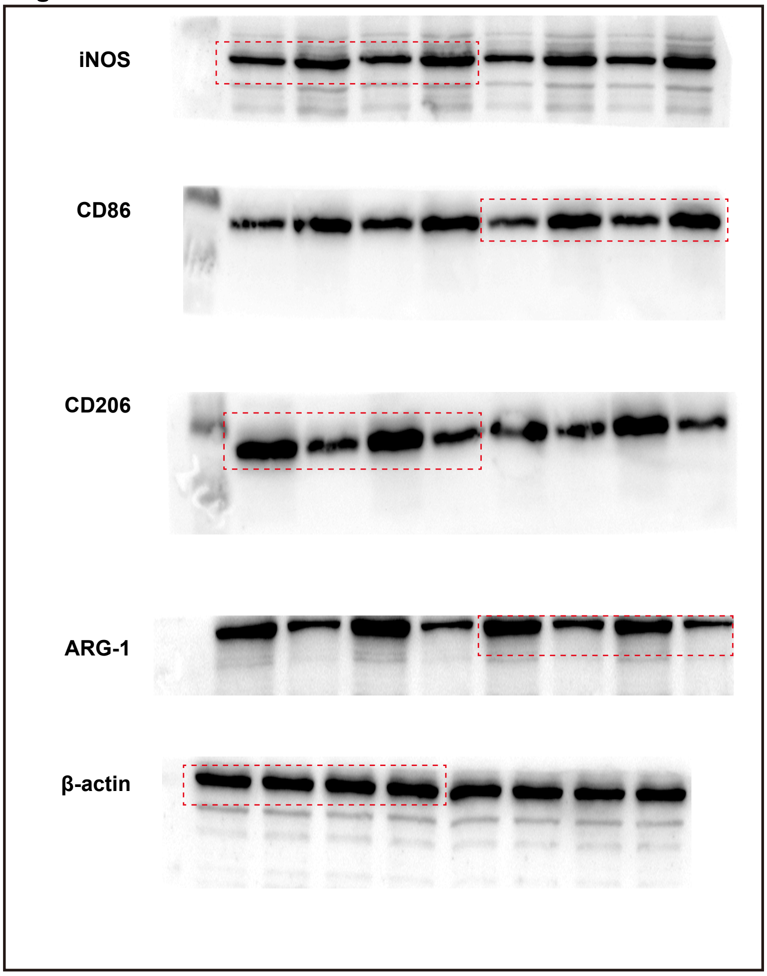

Supplement: Supplementary file 2 — Original Data File [file 41419_2023_6173_MOESM2_ESM.pdf]
